# Digital tools to support mental health: a survey study in psychosis

**DOI:** 10.1186/s12888-023-05114-y

**Published:** 2023-10-07

**Authors:** Emily Eisner, Natalie Berry, Sandra Bucci

**Affiliations:** 1 Division of Psychology and Mental Health, School of Health Sciences, Faculty of Biology, Medicine and Health, Manchester Academic Health Sciences, The University of Manchester, 2nd Floor Zochonis Building, Brunswick Street, Manchester, M13 9PL UK; 2 Greater Manchester Mental Health NHS Foundation Trust, Manchester, UK

**Keywords:** Psychosis, Schizophrenia, Digital health, Survey, Smartphone, Wearable

## Abstract

**Background:**

There is a notable a gap between promising research findings and implementation of digital health tools. Understanding and addressing barriers to use is key to widespread implementation.

**Methods:**

A survey was administered to a self-selecting sample in-person (*n* = 157) or online (*n* = 58), with questions examining: i) ownership and usage rates of digital devices among people with psychosis; ii) interest in using technology to engage with mental health services; and iii) facilitators of and barriers to using digital tools in a mental healthcare context.

**Results:**

Device ownership: Virtually all participants owned a mobile phone (95%) or smartphone (90%), with Android phones slightly more prevalent than iPhones. Only a minority owned a fitness tracker (15%) or smartwatch (13%). Device ownership was significantly lower in unemployed people and those without secondary education. Device cost and paranoid ideation were barriers to ownership.

Technology and mental health services: Most participants (88%) said they would willingly try a mental health app. Symptom monitoring apps were most popular, then appointment reminders and medication reminders. Half the sample would prefer an app alongside face-to-face support; the other half preferred remote support or no other mental health support.

Facilitators: Participants thought using a mental health app could increase their understanding of psychosis generally, and of their own symptoms. They valued the flexibility of digital tools in enabling access to support anywhere, anytime.

Barriers: Prominent barriers to using mental health apps were forgetting, lack of motivation, security concerns, and concerns it would replace face-to-face care. Overall participants reported no substantial effects of technology on their mental health, although a quarter said using a phone worsened paranoid ideation. A third used technology more when psychotic symptoms were higher, whereas a third used it less. Around half used technology more when experiencing low mood.

**Conclusions:**

Our findings suggest rapidly increasing device ownership among people with psychosis, mirroring patterns in the general population. Smartphones appear appropriate for delivering internet-enabled support for psychosis. However, for a sub-group of people with psychosis, the sometimes complex interaction between technology and mental health may act as a barrier to engagement, alongside more prosaic factors such as forgetting.

**Supplementary Information:**

The online version contains supplementary material available at 10.1186/s12888-023-05114-y.

## Background

Over the past decade, countless digital health tools for people with severe mental health problems (SMI) have been developed and tested worldwide [1–5]. Numerous studies have demonstrated high rates of engagement with digital health tools among people with schizophrenia-spectrum psychosis (hereafter shortened to ‘psychosis’), including samples at high risk of relapse [6–9], and evidence of efficacy and effectiveness is rapidly accumulating [10, 11]. Such advancements could help address challenges that services and service users face in delivering and receiving time-sensitive healthcare. However, there is a notable a gap between promising research findings and implementation of digital health tools in services. Compared with other mental health groups, people with psychosis are often excluded from accessing digital health technologies [12].

A range of barriers, including very practical barriers (e.g. owning a device), can hinder successful implementation of digital tools. Research in psychosis samples examining implementation barriers and facilitators is ongoing [13]. Although several studies have examined device ownership in samples with psychosis, the evolving landscape of ever-increasing ownership, globally, means these figures need updating regularly.

A meta-analysis of studies published 2009–2015 showed an overall mobile phone ownership rate among people with psychosis of 66.4% [14]. In a scoping search, we found ten studies [15–24], published since this meta-analysis [14], that reported device ownership among people with psychosis: four from the US, two from the UK (London only), and four from elsewhere (Spain, Portugal, Australia, Canada). We calculated that the average mobile phone ownership rate among these studies, weighted by sample size, was 82% (range 60%-98%), with the weighted average for smartphone ownership being 62% (range 54%-84%). Evidently there was a marked increase in device ownership in the intervening years.

Even studies published the same year as the meta-analysis [14] report substantially higher usage, emphasising how rapidly this increased. For example, Gay and colleagues [15] reported 82.9% mobile phone access, 54% smartphone access and 89% computer access, and a UK study [16] reported 54% smartphone ownership. The latter study also reported that only 18% of the sample were digitally excluded in 2016, compared to 30% in 2011.

As smartphone and other device ownership rates continue to increase, and often vary geographically, little is known about current rates of device ownership and use among people with psychosis in the UK, and how individuals with psychosis interact with and feel about digital devices. Understanding and addressing barriers to device use, in order to close the ‘digital divide’, is key to widespread implementation and adoption of digital health strategies.

The aims of this survey study are to: i) understand digital device ownership and usage rates in a sample of people with psychosis; ii) explore interest in using technology to engage with mental health services; and iii) understand facilitators of and barriers to using digital tools in the context of mental healthcare.

## Methods

### Setting and participants

The survey was conducted between April 2018 and September 2020. Individuals enrolled in a digital health trial in secondary care services in the Northwest of England, Actissist2 (Bucci S: Effects of Actissist, a digital health intervention for early psychosis: a randomized clinical trial, in preparation), or who responded to online advertisements, were invited to take part in this survey study. Inclusion criteria were: i) schizophrenia-spectrum diagnosis (determined by a clinician, if recruited through Actissist2, or self-reported if recruited online) or meeting criteria to receive care for a psychosis-related disorder from secondary care mental health services; ii) aged > 16 years; iii) able to provide consent; iv) English speaking.

The survey was administered in paper-based format or via online survey software. Paper-based surveys were completed in a researcher’s presence, with support if required (e.g. reading items if poor eyesight or reading difficulties). The online version was administered via REDCap [25, 26]. Advertisements were posted in mental health service waiting areas, on mental health charity websites, University research pages, and social media (e.g. Twitter). The survey took approximately 20 min to complete and was approved by the relevant ethics committee. Participants received written information about the study, indicated consent before proceeding and could enter a prize draw (£50 prize).

### Survey design and development

Data were collected through a cross-sectional survey gathering information about participants’ use of and views about digital technology (see Additional file 1), alongside basic demographic information. We reviewed and included survey items from other studies [16, 27–32] and included our own items informed by qualitative study findings [33–36]. Items were generally rated on Likert scales reflecting level of agreement or frequency of use. Patient and public contributors reviewed survey items for acceptability and relevance.

### Data analysis

Descriptive statistics explored technology ownership/usage, interest in mental health apps, and attitudes towards technology. Relationship between survey responses and demographic variables were examined using Mann–Whitney U test (non-normally distributed continuous variables), Chi squared test (categorical variables) or Fisher’s exact test (categorical variables with expected cell counts < 5 [37]; i.e., mobile ownership, smartphone ownership).

We excluded 26 participants who consented but completed no survey items. In presenting data from the remaining participants, we dealt with missing data by excluding cases listwise. Analyses were conducted using Stata Version 14.0 [38]. Statistical tests were considered statistically significant at *P* < 0.05. Where possible, bootstrapping produced 95% confidence intervals.

## Results

### Respondent characteristics

In total, 215 people completed the survey: 73% (*n* = 157) in-person in the context of the Actissist2 digital health trial, and 27% (*n* = 58) online. Table 1 presents demographic characteristics for the two sub-samples and overall sample. Most participants had a psychosis/schizophrenia diagnosis (psychosis: 131/215, 61%; schizophrenia: 29/215, 13%). Slightly over half were male and the median age was 26 years. Quarter were from ethnic minority groups, most had completed secondary education, and slightly under half were in paid/voluntary employment or education.

**Table 1:** Demographic characteristics of the sample (*n* = 215)

	Actissist2 sample (*n* = 157)		Online sample (*n* = 58)		Total sample (*n* = 215)	
	N	%	N	%	N	%
**Age**						
Median (range)	26	(17–54)	26	(17–63)	26	(17 – 63)
**Gender**						
Female	57	36.3	38	65.5	95	44.2
Male	100	63.7	17	29.3	117	54.4
Non-Binary/agender	0	0.0	3	5.2	3	1.4
**Ethnicity**						
White	111	70.7	49	84.5	160	74.4
Asian	23	14.7	1	1.7	24	11.2
Mixed ethnicity	9	5.7	5	8.6	14	6.5
Black	11	7.0	0	0.0	11	5.1
Arabic	2	1.3	0	0.0	2	0.9
Other ethnic group	1	0.6	3	5.2	4	1.9
**Employment status**						
Employed	27	17.3	29	50.9	56	26.3
Voluntary work	8	5.1	0	0.0	8	3.8
Student or apprentice	20	12.8	13	22.8	33	15.5
Unemployed	98	62.8	15	26.3	113	53.1
Household/caring duties	3	1.9	0	0.0	3	1.4
**Education completed**						
Primary or less	8	5.1	0	0.0	8	3.7
Secondary	95	60.9	35	60.3	130	60.8
Tertiary/further	52	33.3	23	39.7	75	35.1
Unsure/rather not say	1	0.6	0	0.0	1	0.5
**Relationship status**						
Single	115	73.2	26	46.4	141	66.2
In a relationship	10	6.4	18	32.1	288	13.1
Cohabiting	15	9.6	3	5.4	18	8.5
Married/civil partnership	13	8.3	6	10.7	19	8.9
Divorced/separated	4	2.6	3	5.4	7	3.3
**Diagnosis**						
No diagnosis received	8	5.2	7	12.1	15	7.0
Schizophrenia/ psychosis	134	86.5	26	44.8	160	75.1
Schizoaffective	3	1.9	11	19.0	14	6.6
Bipolar	3	1.9	6	10.3	9	4.2
Other	7	4.5	8	13.8	15	7.0

The two subsamples differed significantly in gender (χ^2^(2) = 25.50, *P* = 0.001; Actissist2 subsample more male), ethnicity (χ^2^(5) = 17.45, *P* = 0.004; Actissist2 more ethnically diverse), relationship status (χ^2^(4) = 27.47, *P* = 0.001; Actissist2 more single) and diagnosis (χ^2^(3) = 18.21, *P* = 0.001; Actissist2 sample more schizophrenia/psychosis diagnoses). Subsamples did not differ in age (z = -0.304, *P* = 0.76), education (χ^2^(2) = 3.83, *P* = 0.15) or employment status (χ^2^(3) = 3.83, *P* = 0.28).

### Device ownership and use

#### Mobile phones

**Fig. 1:**
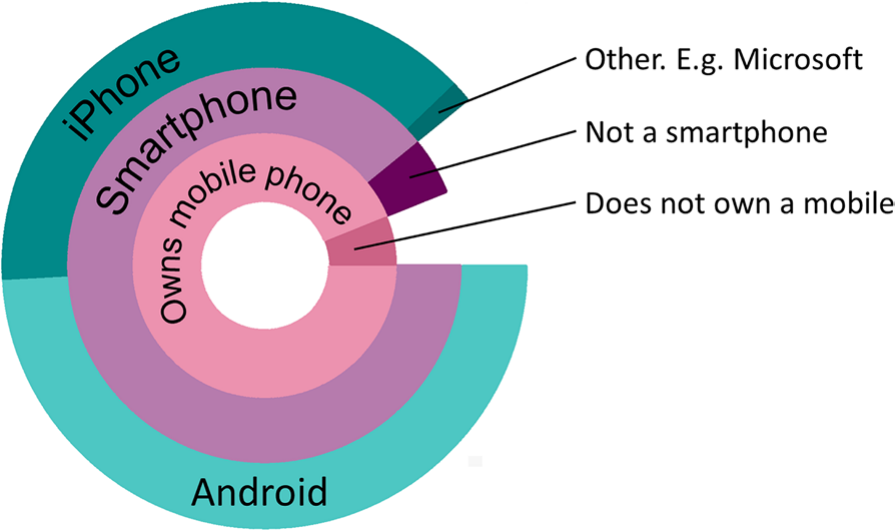
Breakdown of mobile phone ownership by device type. Starting from the centre, the three circles represent the whole sample (*N* = 215, pink), mobile phone owners (*n* = 204, purple) and smartphone owners (*n* = 194, turquoise), respectively

Almost all participants owned a mobile phone (95%) and most (90%) had smartphones (of which: 55% were Android, 44% iOS, 1% other; Fig. 1). Of 11 participants without their own phone, nine had occasional access to someone else’s (5 Android, 2 iOS, 1 unknown, 1 non-smartphone).

Unemployed people were significantly less likely to own any type of mobile phone (unemployed 92%; others 98%; *P* = 0.046), and significantly less likely to own a smartphone specifically (unemployed 89%; others 97%; *P* = 0.016), than people in paid/voluntary work, who were a student/apprentice, or who had household/caring duties. Similarly, people whose highest education was primary school were less likely to own any type of mobile phone (primary education 75%; secondary 96%; tertiary 95%; *P* = 0.07), and less likely to own a smartphone specifically (primary 63%; secondary 94%; tertiary 95%; *P* = 0.02), than those with secondary/tertiary education, although this only reached statistical significance for smartphone ownership. Mobile phone and/or smartphone ownership rates were not significantly associated with any other participant characteristics (gender, ethnicity, relationship status, diagnosis or age). However, Actissist2 participants were significantly less likely to own a mobile phone than online participants (Actissist2 93%; online 100%; *P* = 0.04), but there was no significant difference in smartphone ownership (Actissist2 91%; online 97%; *P* = 0.25).

Regarding paying for phone use, 34% had a Pay-As-You-Go phone, 59% a monthly contract (37% phone-plus-SIM contract; 22% SIM-only), and 7% reported that someone else paid for their phone use. Around 42% (90/212) endorsed at least one barrier to owning/using a mobile phone, including: feeling paranoid/suspicious about mobile phones (*n* = 42), struggling to afford one (*n* = 29), repeatedly losing/damaging them (*n* = 28), not knowing how to use certain phone features (*n* = 15), lack of interest (*n* = 10) or need (*n* = 7) for a mobile phone, and not knowing how to use one (*n* = 3).

**Fig. 2:**
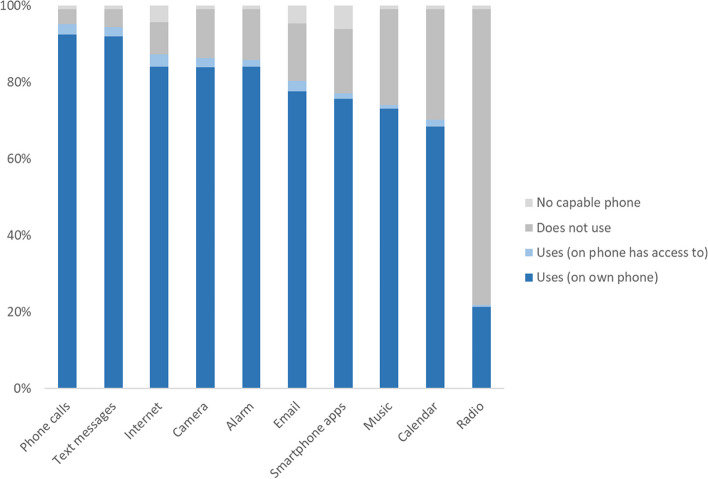
Percentage of participants who report using specific mobile phone features, either on their own phone or someone else’s phone which they had access to

Figure 2 shows the percentage of participants reporting using specific features of their mobile phone (or one they had access to). The features most respondents used were making phone calls (95%), sending text messages (94%), internet (87%), camera (86%) and alarm (86%). Most respondents also used a mobile phone for email (80%), apps (77%), music (74%), and the calendar (70%), while only a small proportion (22%) used a phone for the radio.

#### Wearable devices

A minority (21%) owned a wearable device (15% fitness tracker; 13% smartwatch), with an additional 3% reporting access to a fitness tracker (not owned). Unemployed people were significantly less likely to own a fitness tracker (unemployed 7%; others 23%; *P* = 0.001) or smartwatch (unemployed 7%; others 19%; *P* = 0.02). People with a schizophrenia/psychosis diagnosis were less likely to own a fitness tracker (schizophrenia/psychosis 12%; other 29%; *P* = 0.02) or smartwatch (schizophrenia/psychosis 10%; other 26%; *P* = 0.02). Ownership rates for fitness trackers, but not smartwatches, were significantly lower among single people (10% owned a fitness tracker) than people in a relationship (23% owned one; *P* = 0.02) and among the online sample (31%) than the Actissist2 sample (9%; *P* = 0.001). Gender, ethnicity, education and age were not significantly associated with ownership rates of either device.

#### Frequency of phone and other device use

**Fig. 3:**
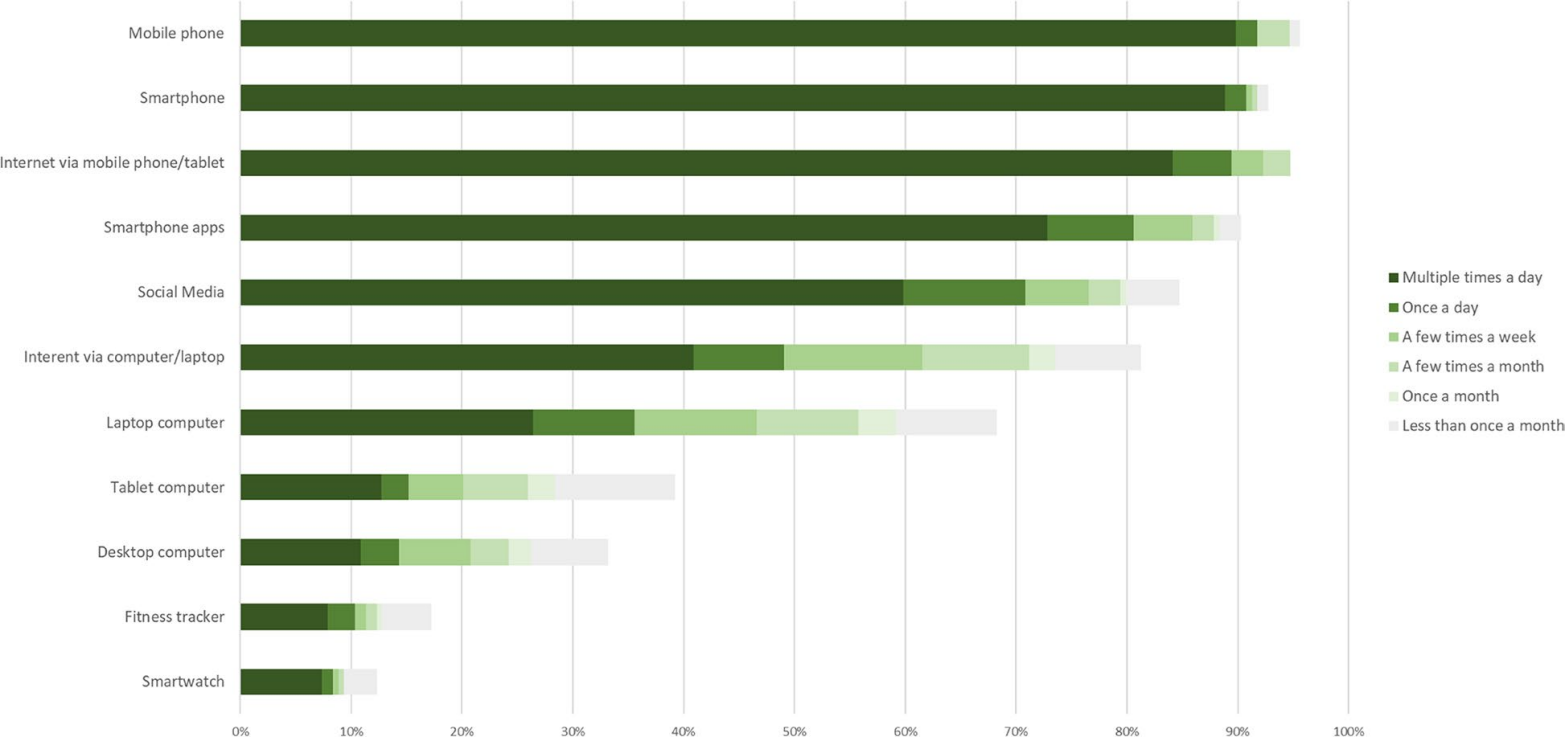
Frequency of device use

As Fig. 3 shows, > 80% of respondents used a mobile phone/smartphone and the internet (via mobile phone/tablet) multiple times a day. Around two-thirds also used apps and/or social media multiple times a day, with most using these features at least once a month. Over half of respondents used a laptop at least once a month (59%), with only a third using laptops daily. Most used fitness trackers infrequently, with a very small minority using a fitness tracker (11%) or smartwatch (9%) multiple times a day/daily/a few times a week.

### App and social media use

#### App use

**Fig. 4:**
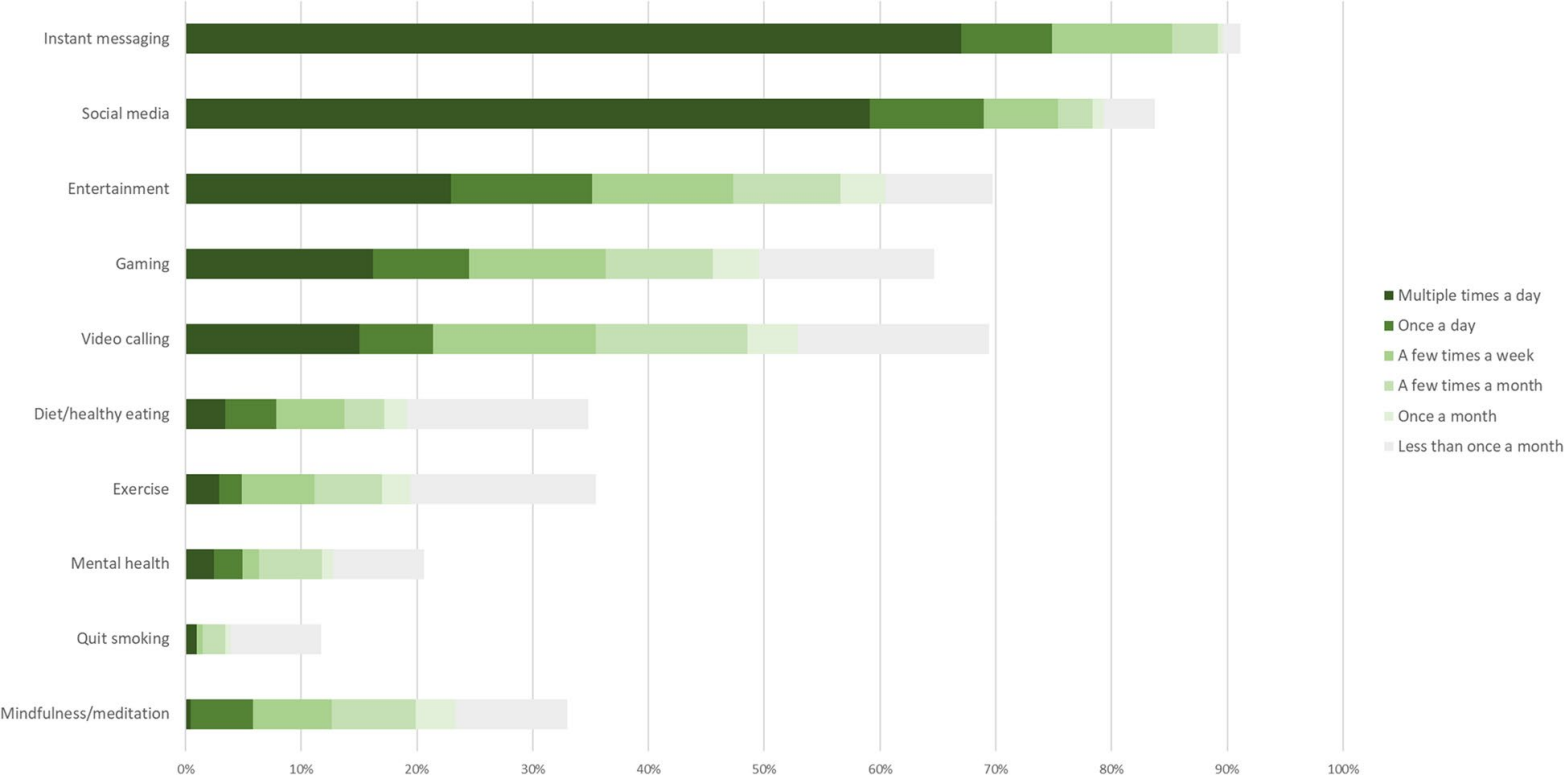
Frequency of smartphone app use

Figure 4 shows frequency of smartphone app usage. Most respondents used instant messaging and social media apps multiple times a day. Around a third used entertainment apps at least daily. Less frequently used apps were gaming, video calling and health-related apps.

**Fig. 5:**
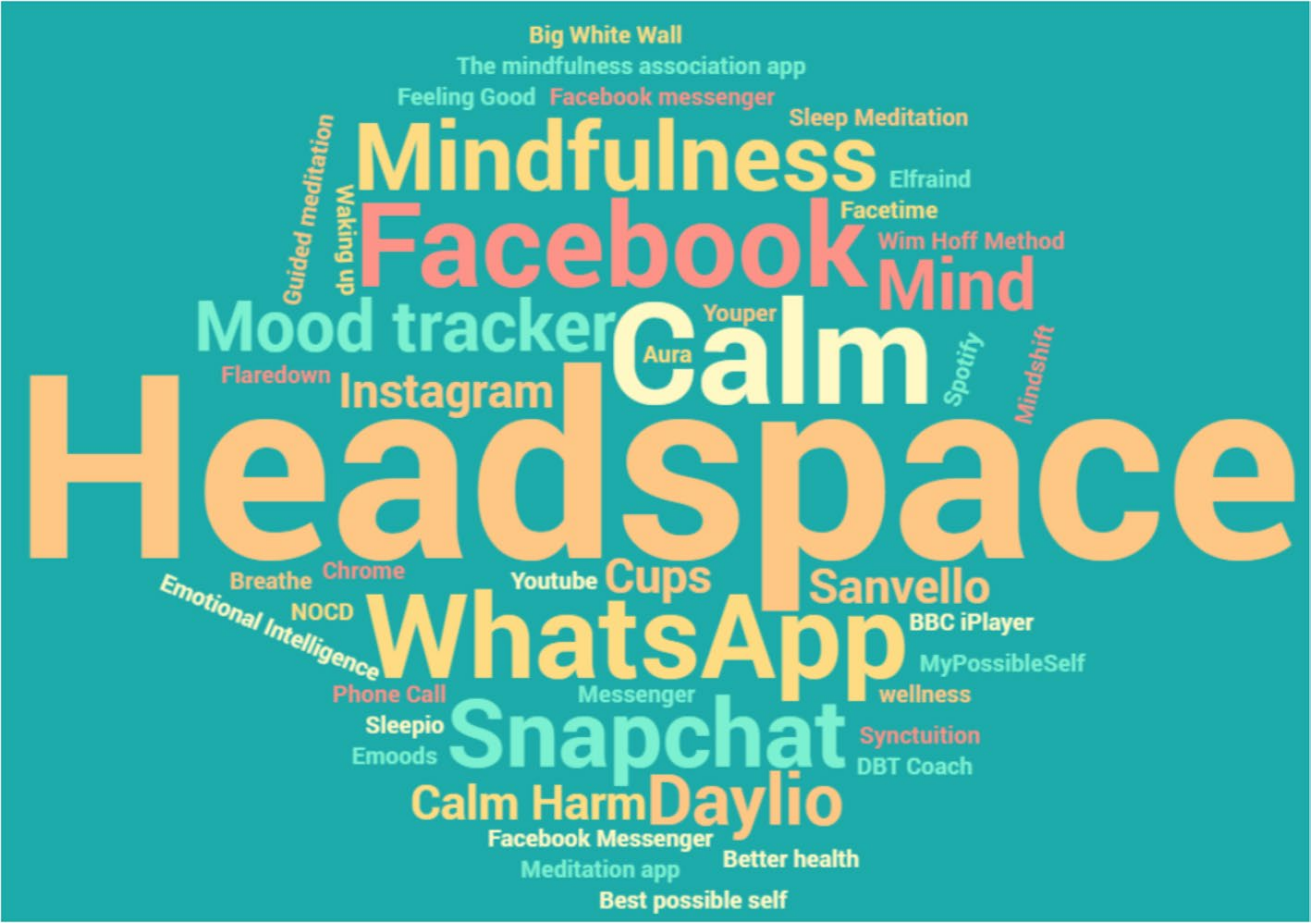
Word cloud to illustrate the range of apps participants listed in response to the instruction: “If you have ever used mental health/wellbeing/mindfulness app(s), please indicate which apps these were”

Figure 5 illustrates the apps participants listed in response to the instruction “If you have ever used mental health/wellbeing/mindfulness app(s), please indicate which apps these were”. Headspace was the most listed health app, with other frequently listed health apps including Mindfulness, Calm, and Mood Tracker. Participants also listed more general apps in response to this question, including social media apps, presumably because they used these to support their mental health. Of app use reported in response to this question, 49% was current use (51% past), with 44% at least daily, 34% at least monthly and 22% less than monthly. Slightly under half (46%) of this app use was judged helpful/very helpful, with 43% neutral and 11% “unhelpful – made me feel worse”.

#### Social media use

**Fig. 6:**
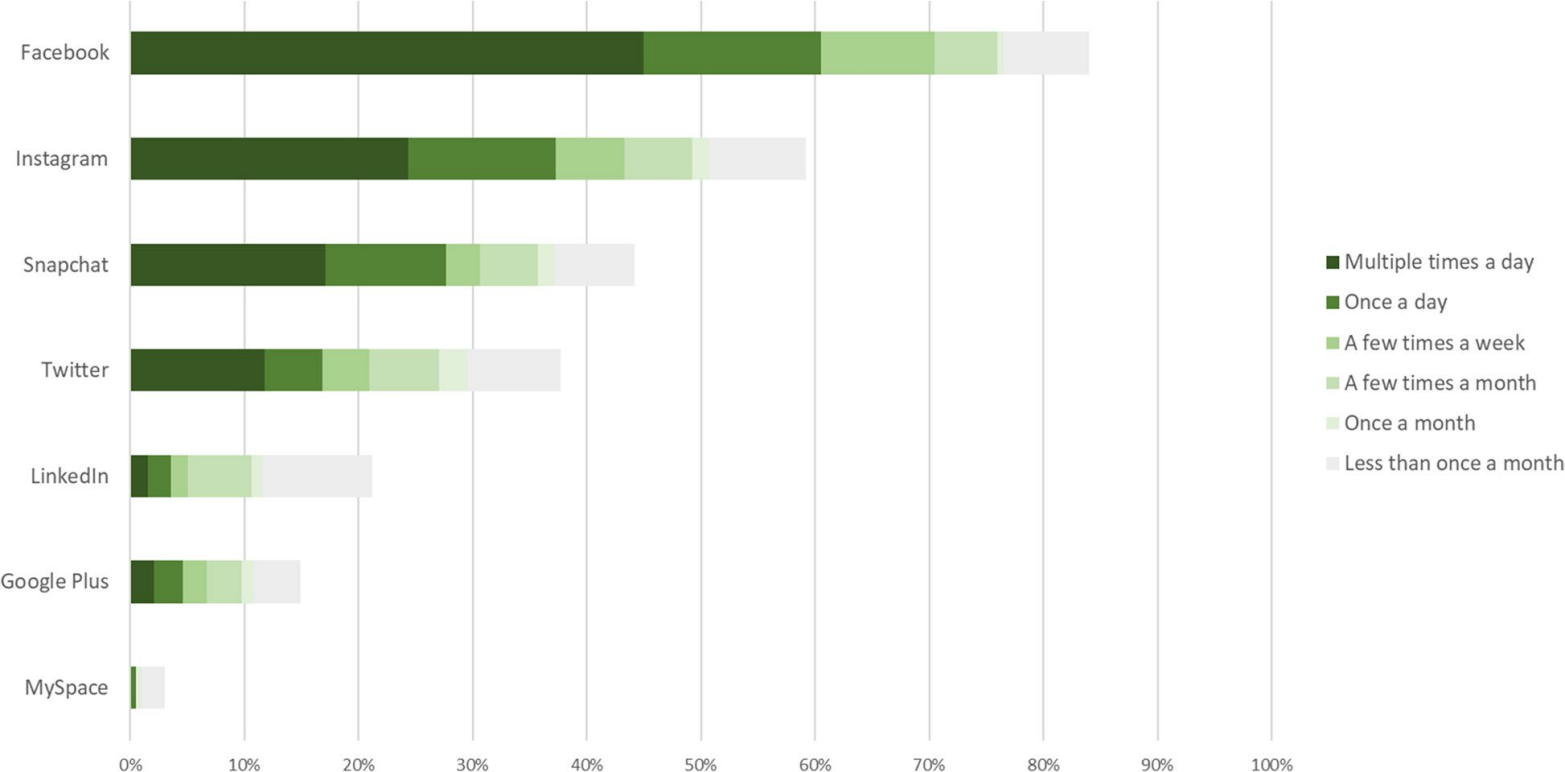
Frequency of using social media sites

Overall, 85% reported using social media at some point. Unemployed people were significantly less likely to use social media than others, but no other demographic variables were associated with use (Table 2). Figure 6 shows frequency of using specific social media sites. Facebook was the most popular (71% used a few times a week), followed by Instagram (43%), Snapchat (31%) and Twitter (21%). Very few ever used LinkedIn (21%), Google Plus (15%) or MySpace (3%).

**Table 2:** Associations between demographic variables and the likelihood of being a social media user and (for social media users only) the likelihood of posting on social media about physical or mental health. Unless otherwise specified, Fisher’s exact test was used

	Social media user		Physical health posts		Mental health posts	
	%	*P*	%	*P*	%	*P*
**Age**^**a**^						
(z, *P*)	1.657	.10	-1.578	.12	-0.091	.93
**Gender**						
Female	87.4	.34	37.5	.02	37.5	.07
Male	82.0		19.5		24.1	
**Ethnicity**						
White	85.9	.58	33.3	.008	35.7	.003
Mixed ethnicity	85.7		33.3		41.7	
Asian, Black, Arabic, other ethnic group	79.5		6.9		6.9	
**Employment**						
Unemployed	79.1	.04	79.09	.03	26.5	.47
Employed	87.3		87.27		35.6	
Voluntary work, student, apprentice, household or caring duties	95.2		95.24		35.0	
**Education**						
Primary or less	85.7	.99	0.0	.46	0.0	.29
Secondary	85.0		30.5		34.3	
Tertiary/further	84.9		28.8		28.8	
Unsure/rather not say						
**Relationship**						
Single	83.7	.34	21.3	.02	23.2	.02
Relationship, cohabiting, married or civil partnership	87.7		41.1		44.6	
Divorced or separated	71.43		50.0		25.0	
**Diagnosis**						
Schizophrenia/psychosis	84.0	.81	24.3	.01	27.2	.02
Other diagnosis	87.2		48.5		48.5	
**Sample**						
Online sample	92.7	.08	53.2	< .001	66.0	< .001
Actissist2 sample	81.8		19.5		17.9	

^a^ Mann Whitney U test. Z and *P* values are provided rather than % and *p* values

Nearly half (48%) of social media users would like to participate in a social media group of others with psychosis. Demographic characteristics did not distinguish those who would vs would not like to participate. Less than a third of participants had previously posted on social media about their physical health (29%) or mental health (31%). There were several statistically significant associations with demographic characteristics (Table 2), including: gender (females posted most), ethnicity (white and mixed race people posted most), employment (employed/volunteers/students and people with household/caring duties posted most), relationship status (people in a relationship posted most) and diagnosis (people with a schizophrenia/psychosis diagnosis posted least). Finally, online participants were significantly more likely to have posted about physical or mental health than Actissist2 participants.

### Technology and engagement with services

#### Interest in mental health app

Most (88%) would try a “mobile phone app for mental health” if offered (8% unsure). Actissist2 participants were significantly more likely to want to use one (93% would use) than the online sample (71% would use; *P* = 0.001). Of participants expressing interest in using one, around half (53%) would want to use a mental health app in conjunction with face-to-face support, 14% with remote mental health support and 32% on its own without other mental health support. Support preferences did not differ significantly between Actissist2 participants and online participants (*P* = 0.24). Around half of interested participants (47%) would not be willing to pay for a mental health app; 24% would pay up to £0.99 and 28% would pay more than £0.99 for such an app.

#### Symptom monitoring apps

As with a more general “mental health app”, most participants (86%) would use a phone app to record their symptoms over time. Of those interested, 67% were willing for symptom data to be automatically transferred to the care team, 17% would want to choose which information was transferred, and 10% would like to show the care team their app responses directly during an appointment. Only 6% would not want their care team to see the app-reported symptom information. Actissist2 participants were more interested in automatic transfer (75% endorsed this option) than online participants (36%; *P* = 0.001). Regarding how the app asks symptom questions, 46% preferred app-generated reminders, 19% preferred to answer when they chose, and 35% would like a combination. Actissist2 participants were significantly more likely to want reminders (49%) than online participants (36%; *P* = 0.04), who tended to prefer a combination of reminders and free choice.

#### Text messaging vs. apps

**Fig. 7:**
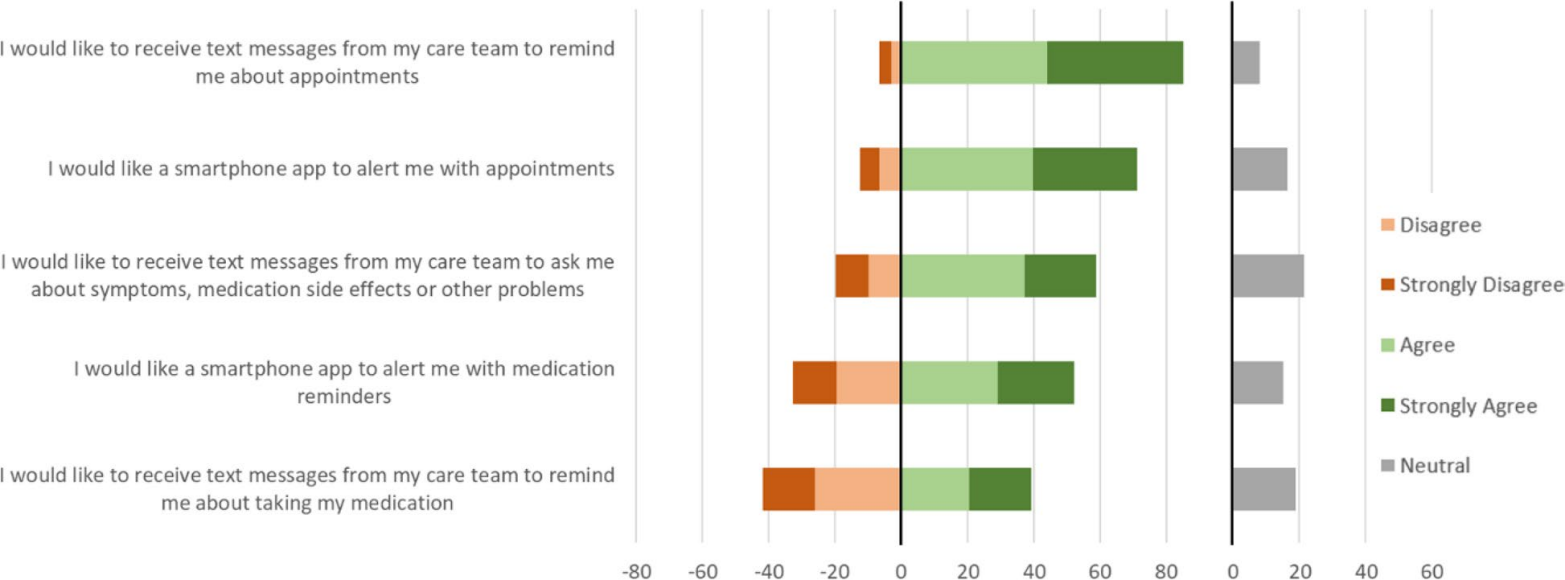
Participants’ preferences for receiving text messages or smartphone app alerts from the care team

Fewer participants (59%) were interested in receiving text messages from their care team asking about “symptoms, medication side effects or other problems” (Fig. 7) than were interested in phone apps for a similar purpose (described above). Regarding preferences for receiving smartphone app alerts or text messages from their care team (Fig. 7), appointments reminders were popular (71% interested in receiving app alerts; 85% text messages) and medication reminders less popular (52% interested in app alerts; 39% interested in text messages).

#### Perceived barriers to mental health app use

**Fig. 8:**
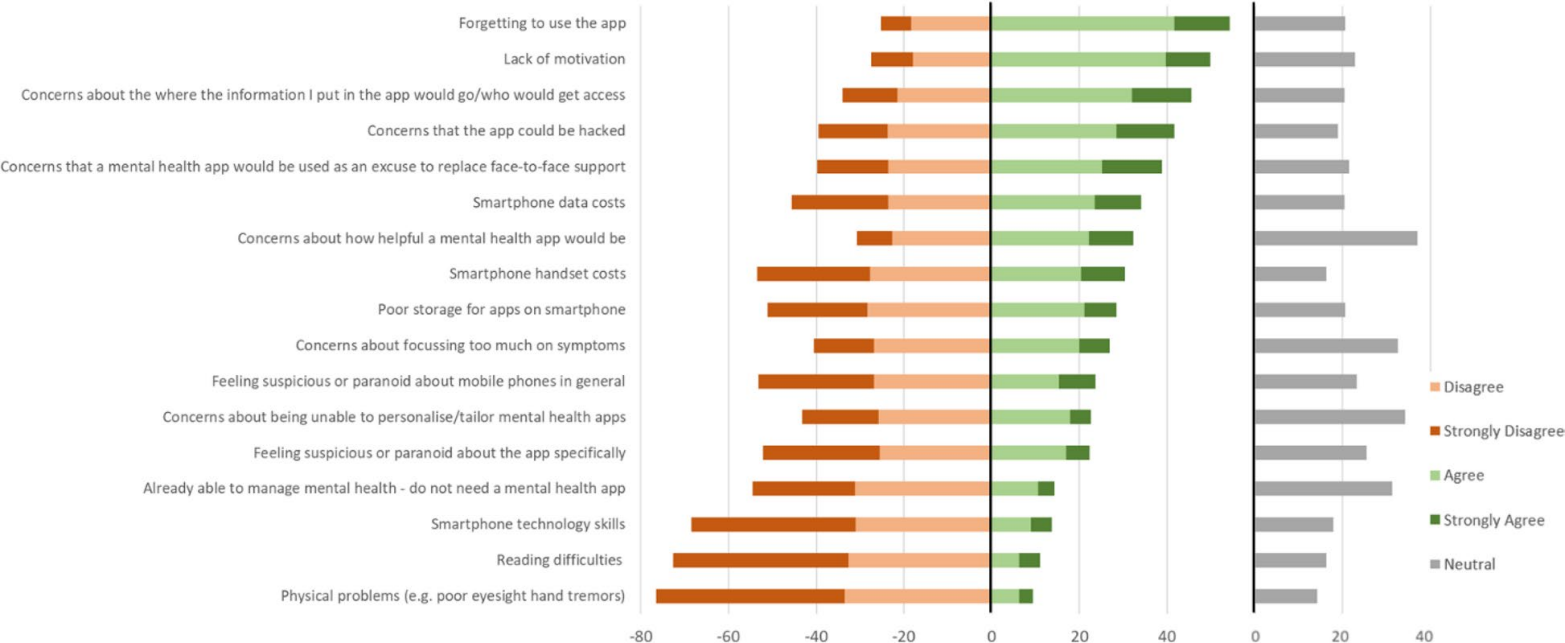
Perceived barriers to mental health app use

As Fig. 8 shows, the most frequently endorsed barriers (rated “agree” or “strongly agree”) to using a mental health app were forgetting (54% of participants) or lacking motivation (50%) to use an app. These were followed by data security concerns about who would access the information (46%) or the app being hacked (42%), and concerns that mental health apps might replace face-to-face support (39%). Other barriers were endorsed by less than 35% of participants. The least endorsed were smartphone technology skills (14%), reading difficulties (11%) and physical problems (9%). A content analysis of free-text responses (*n* = 36) yielded several additional barriers, including lack of time to use an app due to work/life commitments (*n* = 7), additional costs such as app subscription or electricity (*n* = 4), and poor phone battery (*n* = 3).

#### Perceived advantages of mental health apps and suggested additional content

**Fig. 9:**
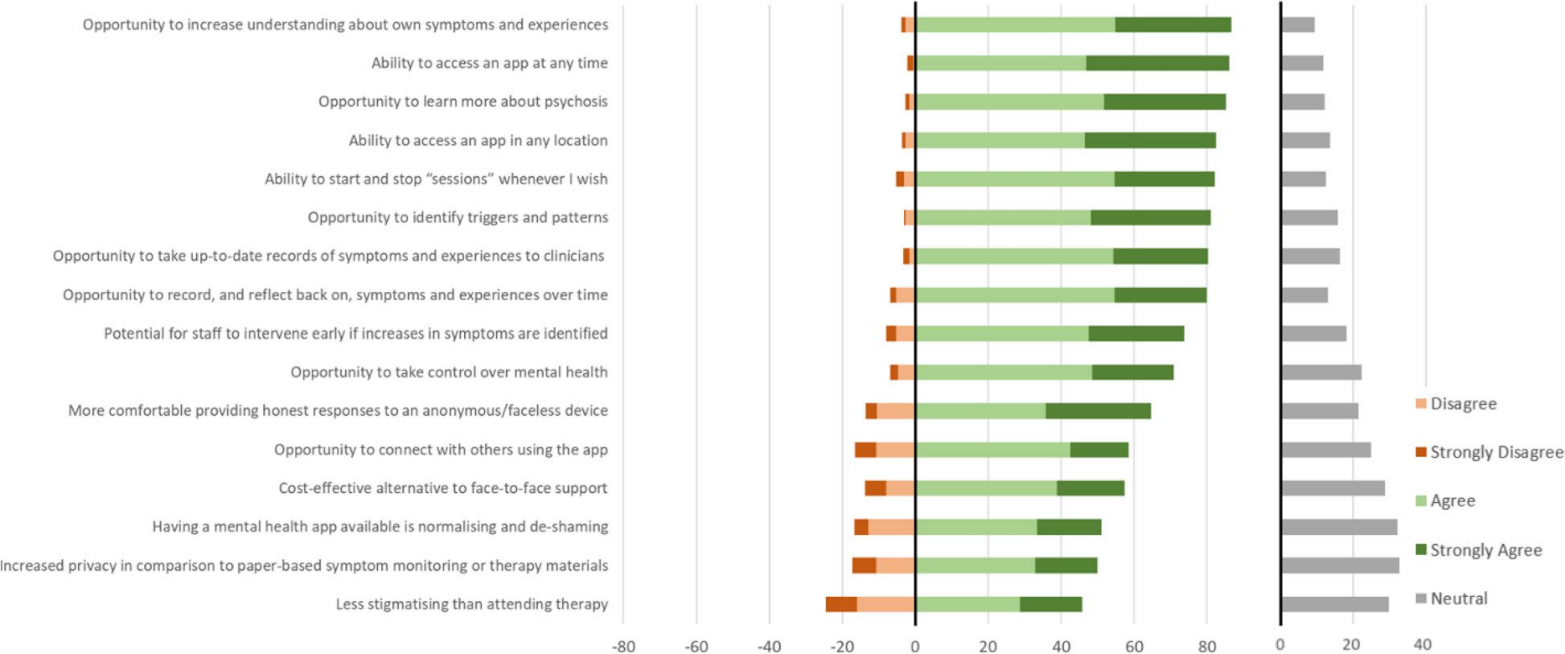
Perceived advantages of mental health app use

As Fig. 9 shows, two frequently endorsed advantages (rated “agree” or “strongly agree”) of mental health apps related to increasing one’s understanding of one’s own symptoms (87%), and of psychosis more generally (85%). Participants also agreed particularly strongly that an app’s flexibility was an important advantage: being able to access the app at any time (86%) and in any location (83%).

Almost all statements about potential advantages of mental health apps were endorsed by the majority. The one exception was that an app would be “less stigmatising than therapy”.

Participants could state “other reasons why you might want to use a mental health app” (*n* = 59 responded) and “ideas for any other content you would like to see in a mental health app” (*n* = 34) via free-text response. A content analysis is provided in Table 3. Some of these free text responses elaborate on perceived advantages presented in Fig. 9. For example, "recording symptoms in the moment" and "it can remind someone to check their mental health regularly" both highlight key advantages of app-based self-monitoring.

**Table 3:** Content analysis of participants’ free text responses about other reasons for using a mental health app and ideas for any other mental health app content

**Other reason for using a mental health app**	**Frequency**
It might help / it's an extra thing to try	12
To keep track of my mental health (e.g. check progress, spot patterns/triggers)	8
Convenience/ease	5
To take part in research	3
Recording symptoms in the moment, while I remember	3
A place to get thoughts out of my head but not tell them to anyone	2
Could help access support if needed (e.g. notify health professional)	2
Opportunity to take control of mental health	2
It can remind someone to check their mental health regularly	2
Help deal with things in the moment	2
Anxiety about explaining experiences face-to-face to a health professional	2
Self-report more valid than assessment by a doctor	1
Could help me get more organised at ordering medication from GP	1
"NHS are shit"	1
Distraction from symptoms	1
Alternative to face-to-face appointment if can't attend	1
Video calls with mental health workers	1
General Knowledge/Mindfulness	1
An app is normalising	1
To organise my mind	1
Using my phone as a health tool	1
Good for when completing therapy	1
**Ideas for other mental health app content**	**Frequency**
Distraction and calming techniques and resources	4
Online text chat with care team or supportive other	4
Customization (e.g. profile pic, theme, colours)	3
Reminders about personal care (e.g. hygiene, eating, fluid, exercise)	3
Positive statements or reassurance	2
Match me with research studies seeking participants in my area	1
Appointment booking (routine and emergency)	1
A mechanism for posting questions and receiving answers about them	1
A graph to track progress with symptoms	1
Art or music created by people who suffer from mental health issues	1
Drug anonymous page	1
Quick help	1
Things that are important for me to know (for mental health)	1
Very clear information about privacy measures, encryption, data storage, and who has access to data	1
User friendly, with not too much reading required	1

#### Current/previous use of technology for managing mental health

**Fig. 10:**
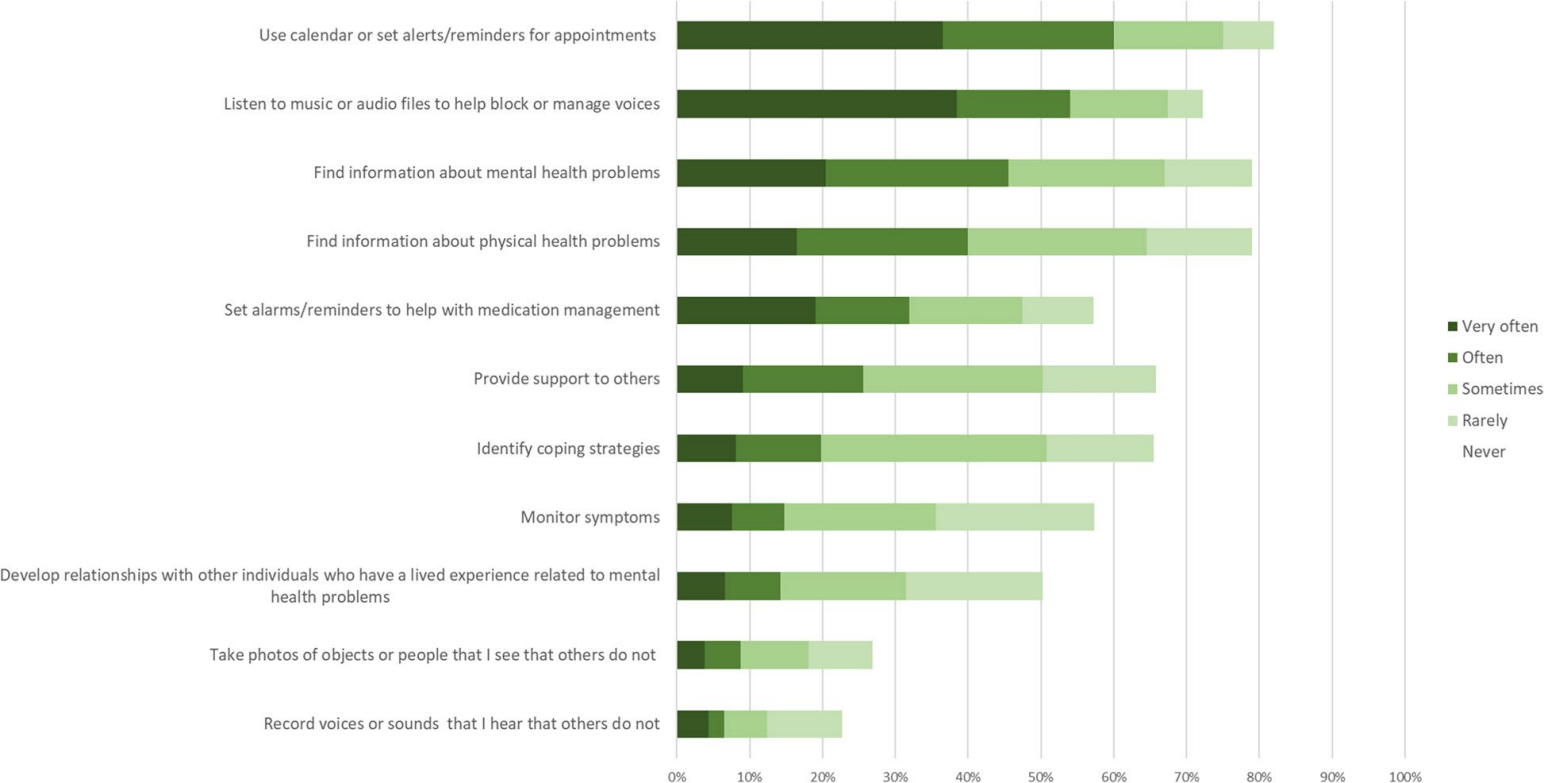
How frequently do you use a computer, mobile phone, smartwatch or tablet computer to do the following?

As Fig. 10 shows, many participants already used technology (mobile phone, smartwatch or computer/tablet) to help manage their mental health. The most popular strategies were using a calendar for appointment reminders (60% “often” or “very often”) and listening to music to block or manage voices (54% “often” or “very often”).

Many participants used technology to find information about mental health problems (46% “often” or “very often”; 79% at some point) or physical health problems (40% “often” or “very often”; 79% at some point). Regarding sharing information with their care team (not shown in Fig. 10), 35% had shared online information about psychiatric medications, and 26% had shared online information about psychological therapy with their care team.

At least half of participants had, at some point, used technology to set reminders about medication, support others, identify coping strategies, monitor symptoms, or connect with others with lived experience of mental health problems. Only a minority used technology for reality testing, e.g., by photographing or recording objects/voices that they see/hear that others do not.

### Beliefs and attitudes about technology

#### Attitudes/enthusiasm towards technology

**Fig. 11:**
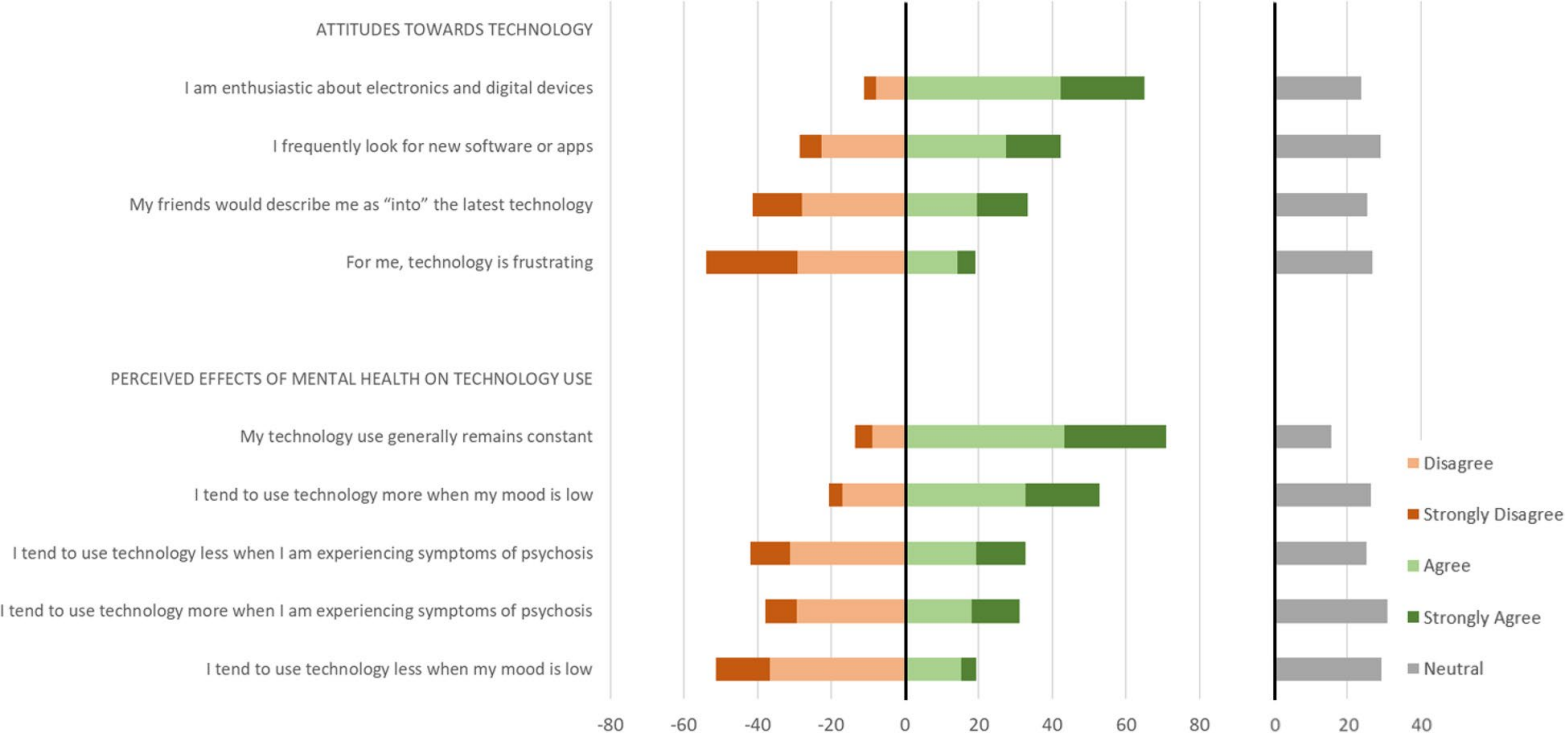
Attitudes towards technology, and perceived effects of mental health on technology use

Although two-thirds of participants were “enthusiastic” about electronics and digital devices, less than half (42%) frequently looked for new software or apps, and only a third would be described by friends as “into” the latest technology (Fig. 11). On the other hand, only a minority (20%) find technology frustrating.

#### Perceived effects of mental health on tech use

Most participants (71%) said their technology use generally remains constant. Nevertheless, some reported changes in technology use depending on their mood or psychotic symptoms (Fig. 11). Around half (53%) used technology more during low mood, whereas 19% used technology less. Regarding psychotic symptoms, 31% used technology more when experiencing symptoms and 33% used technology less.

#### Impact of mobile phone use on socialising, mental health and wellbeing

**Fig. 12:**
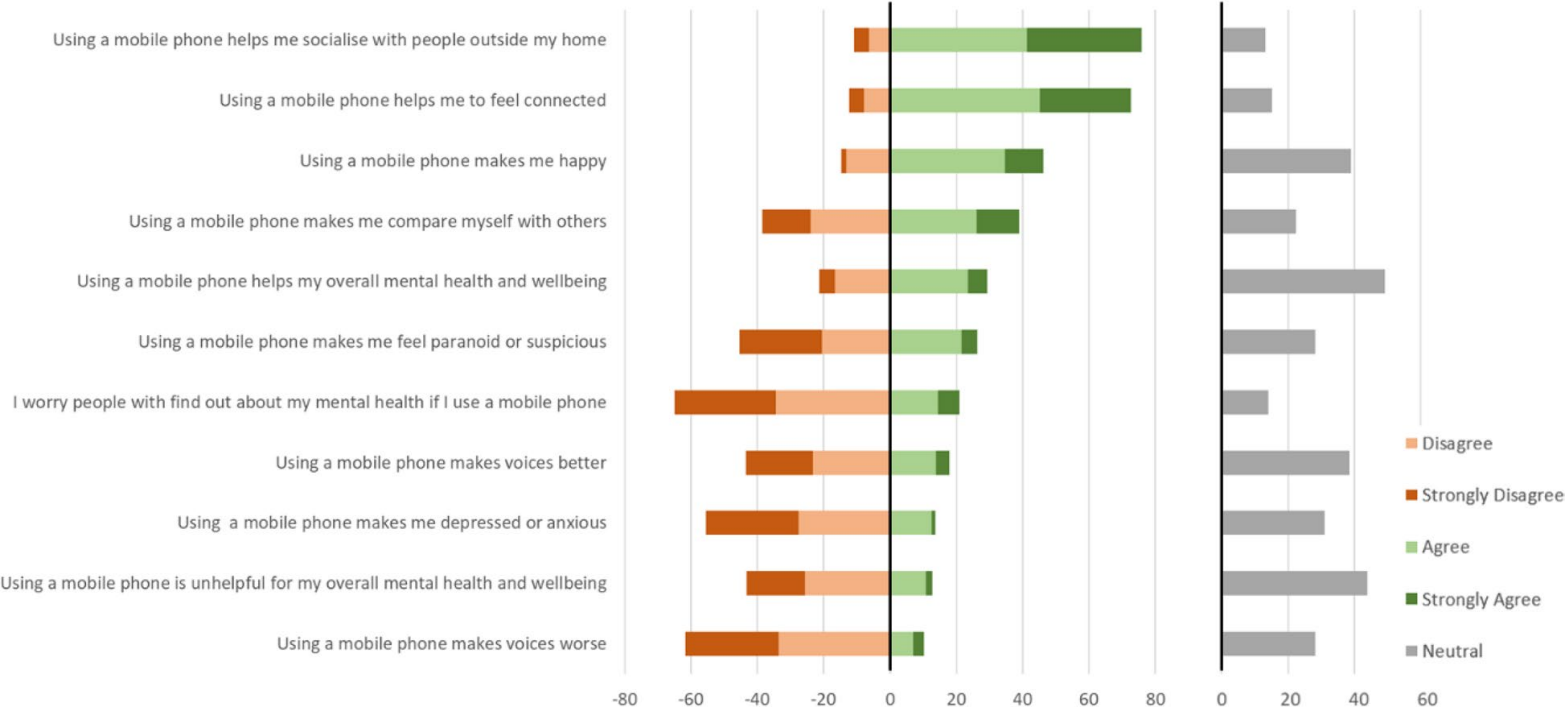
Participants’ beliefs and attitudes about the impact of mobile phone use on socialising, mental health and wellbeing

As Fig. 12 shows, a clear majority reported that using a mobile phone helped them to socialise with people outside their home (76% “agree” or “strongly agree”) and to feel connected (73%). Whilst almost half of participants (46%) agreed or strongly agreed that “using a mobile phone makes me happy”, nearly as many (39%) felt neutral about this statement (only 15% disagreed). Opinions were divided on the idea that “using a mobile phone makes me compare myself with others”, with 39% agreeing and 39% disagreeing (22% neutral); social media users were significantly more likely to agree (44%) than non-social media users (13%; *P* = 0.001).

Generally, participants did not believe that using a mobile phone has a substantial effect on their mental health. Almost half (49%) responded neutrally to the statement “using a mobile phone helps with my overall mental health and wellbeing”, with the rest split roughly evenly between agrees (29%) and disagrees (21%). Similarly, only a minority (13%) found using a mobile phone unhelpful for their overall mental health and wellbeing and only a minority endorsed statements asserting that their mobile phone use made specific symptoms worse (paranoia: 26%; voices: 10%; depression/anxiety: 14%) or better (voices: 18%). Few participants (21%) worried that people would find out about their mental health if they used a mobile phone.

#### Impact of social media use on socialising, mental health and wellbeing

**Fig. 13:**
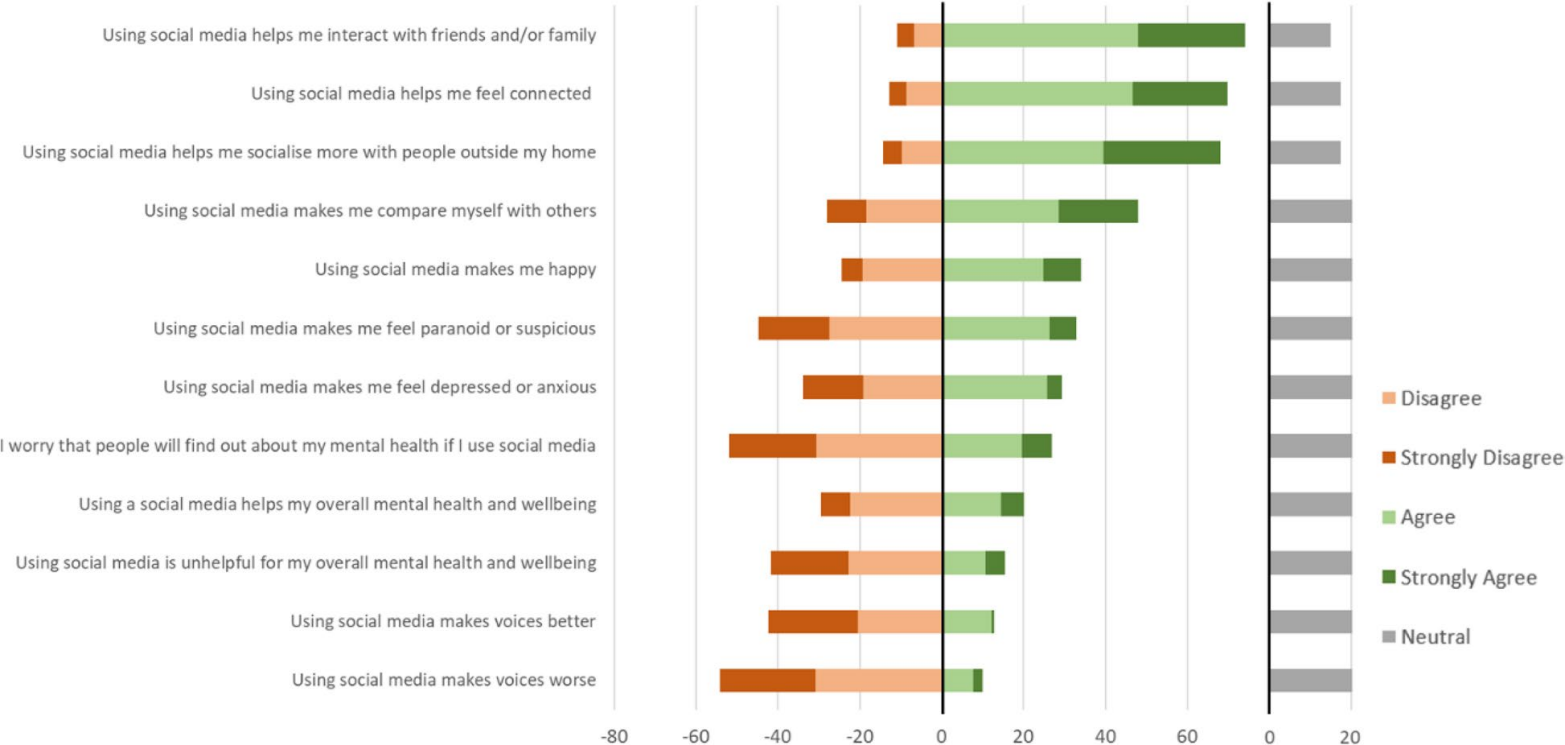
Participants’ beliefs and attitudes about the impact of social media use on socialising, mental health and wellbeing

A similar pattern was seen regarding participants’ beliefs and attitudes about the impact of social media use on their social life, mental health and wellbeing (Fig. 13). As with mobile phones, most participants felt that social media helped them socialise with people outside their home (68%), to feel connected (70%), and to interact with family and friends (74%). The correlations between the equivalent mobile phone and social media variables were 0.64 for socialising (95% Confidence Interval (CI): 0.54–0.75) and 0.59 for connectedness (95% CI: 0.49–0.70).

As with mobile phones, the next two most endorsed statements were around whether social media makes participants compare themselves with others (endorsed by 48%), and whether it makes them happy (endorsed by 34%). Correlations with the equivalent mobile phone variables were 0.60 for comparisons (95% CI: 0.48–0.71) and 0.62 (95% CI: 0.51–0.72) for happiness.

Participants did not generally perceive an effect of social media on their mental health and wellbeing, with only 20% “agreeing” or “strongly agreeing” that social media was helpful, and 16% “agreeing” or “strongly agreeing” that social media was unhelpful. Similarly, regarding specific symptoms, very few participants endorsed the idea that social media made voices better (13%) or worse (10%), and a third or less thought it made them paranoid (33%), or depressed or anxious (29%). As with mobile phones (correlation coefficent 0.59; 95% CI: 0.47–0.70), few participants (27%) were worried people would find out about their mental health if they used social media.

#### Technology 'addiction'

**Fig. 14:**
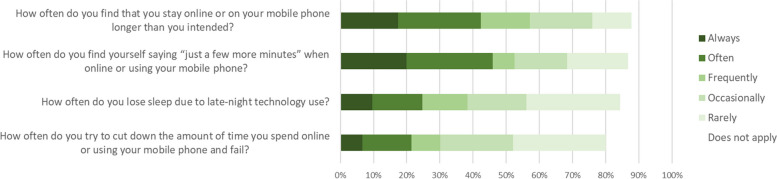
Participants’ responses on four items adapted from the Internet Addiction Test

Items adapted from the Internet Addiction Test indicated that more than half of participants (57%) stayed online or on their mobile phone longer than intended frequently, often, or always (Fig. 14). Similarly, 53% found themselves saying “just a few more minutes” when online or using their phone frequently (or more). Although the impact of late night technology use on sleep was less of an issue, over a third (38%) reported this happened frequently (or more). Only 30% had frequently (or more) tried and failed to cut down the time they spent online or using their mobile phone.

### Other technology

#### Digital pills

Slightly under half of respondents (43%) were “unsure” how helpful digital pills might be for people with psychosis (45% “helpful”; 12% “unhelpful”). Similarly, 45% were “unsure” whether they considered digital pills for psychosis to be acceptable (35% “acceptable”; 20% “unacceptable”). Nevertheless, 40% would be “likely” or “very likely” to use digital pills if they were offered them, with 22% “unsure” (20% “not at all likely”; 12% “unlikely”; 7% not applicable as not taking medication). Of participants who might consider using digital pills, most (86%) would be willing to authorise at least one other person to track their digital pill consumption, with 58% saying this would be their care co-ordinator, 44% their psychiatrist, 41% selected family member(s), 30% their partner, 26% their psychologist and 14% selected friend(s).

#### Artificial agents

A quarter of participants (25%) had communicated with an artificial agent (e.g. iPhone Siri, Microsoft Cortana, Amazon Alexa) about their mental health. Most often, this was to find information about local services (8%), followed by “To feel like I've spoken to someone about how I am feeling” (6%), to find mental health resources (5%) or other reasons (3%). Of those who had communicated with an artificial agent, 61% found the response helpful whereas 33% found it irrelevant and 5% found it upsetting.

## Discussion

This paper describes findings from a UK-based survey exploring ownership and usage rates of digital technologies among a self-selecting sample of people with psychosis, and barriers, facilitators and willingness to engage with services using digital technologies.

### Device ownership

As anticipated, device ownership was higher in our sample (95% mobile phones; 90% smartphones) than previous studies of people with psychosis (weighted averages across ten previous studies: 82% mobile phones; 62% smartphones; [10–18, 23]) or SMI more generally (87% mobile phone ownership; 70% smartphone ownership; [28, 39–47]), likely reflecting the rapid increase in ownership over time and our sample’s relatively young age (median 26 years). Supporting this, a recent general population survey [48] reported 100% mobile phone ownership and 96% smartphone ownership among US adults aged 18–29. The higher prevalence of Android phones than iPhones in our sample is consistent with most previous studies of clinical samples [28, 40, 44], excepting Bell and colleagues’ survey of Australian young people [47]. Wearable device ownership rates (21%) were comparable to recent reports from young people with other mental health diagnoses [47], and the proportion owning fitness trackers (15%) versus smartwatches (13%) mirrored rates observed in the general population approximately two years ago [49].

Taken in the context of previous surveys, our findings suggest that overall device ownership has rapidly increased among people with psychosis, reflecting patterns in the general population. Yet, as others have reported for general mental health samples [45, 47, 50], technology ownership in people with psychosis appears to lag slightly behind the general population. Socio-economic factors may explain this difference. In the general population, device ownership rates tend to increase in line with salary and education [48]. Given that employment and education rates are lower among people with schizophrenia spectrum psychosis than in the general population [51–53], it follows that device ownership might be lower. Indeed, device ownership in our sample was significantly lower in unemployed and less educated people, with device cost an oft-cited barrier to ownership. This said, overall rates of smartphone ownership in our sample were high, implying considerably less digital exclusion than has been previously suggested.

### Engagement with services using digital technologies

Most participants (88%) would be willing to try using a mental health app, which is higher than the interest reported in previous psychosis samples (weighted average 62% interested; [17–19, 24, 54]) or more general mental health samples (72% interested; [42, 44, 47]). The barriers and facilitators for engagement with digital mental health apps in our sample echoed those reported in reviews of general mental health apps (e.g. forgetting, confidentiality [13]) and digital tools for psychosis specifically (e.g. lack of motivation, accessibility [55]).

### Attitudes and beliefs about technology

Users’ attitudes and beliefs about technology are crucial implementation factors [55]. Overall, participants in our sample said technology and social media helped them feel connected to others and the majority reporting no substantial negative effects. Nevertheless, in response to more detailed questions, a third said technology worsened their paranoia, a third increased their technology use when feeling unwell, and a third used it less at such points. Thus, it is important to consider that, for a sub-group of people with psychosis, the sometimes complex interaction between technology use and mental health may be a barrier to engagement. Other studies have highlighted this complexity. In examining the relationship between social media use, mood and paranoia, Berry and colleagues [56] reported that different types of social media use (e.g. posting about feelings vs. daily activities) had different effects on mood and paranoia. Similarly, in a sample with bipolar disorder, technology use was related to symptoms and differed between manic and depressive episodes [32].

Although, as other surveys report [44], participants only rarely used health related apps at present, many already used other smartphone apps to help manage their mental health to some extent (e.g. using music/audio to block voices, calendar for appointment reminders, social media for support). Regarding social media use, people who were female, of white/mixed ethnicity and in a relationship posted most, with unemployed people and people with a schizophrenia/psychosis diagnosis posting least. This echoes findings from a large, nationally representative US survey that female gender, higher education, not being divorced/windowed/separated, and being employed were associated with greater likelihood of posting about health on social media [57]. Around half of social media users would like to participate in a social media group of others with psychosis. A recent survey of Australian young people reported similar levels of interest for connecting with peers about mental health via social media [47].

### Clinical implications and future research

We found high smartphone ownership rates and high interest in mental health apps, suggesting that smartphones are a viable platform for delivering mental health support. Since participants tended to access the internet using a smartphone or tablet more often than a computer/laptop, we recommend that mental health tools are optimised for these formats. Participants generally found the idea of mental health professionals receiving information about their symptoms acceptable, supporting the integration of self-reported mental health data within an individual’s health care record to provide opportunities for early detection of relapse. Nevertheless, despite high ownership rates, 42% of participants endorsed at least one barrier to phone ownership, most commonly paranoia about phones, cost, and repeatedly losing or damaging phone handsets. Clinicians should be mindful of these barriers when considering digital tools as part of the support they provide to individuals with psychosis, particularly in the context of the current cost-of-living crisis [58], which may impede continued increases in device ownership [59].

The fast-paced rate of development and innovation in the digital mental health field means that researchers are now exploring novel ways of providing time-sensitive mental health support, such as ‘passive sensing’ via smartphones/wearables [60]. Some participants in our study reported that their technology use changed in relation to their mental health. As others note [61], such changes in technology use may serve as important biomarkers for relapse, and could be integrated with further digital data to create a “digital phenotype” for relapse prediction, detection and prevention. Prospective research in large samples is needed to identify specific changes in technology use (and other passively collected data) that predict relapse with high sensitivity and specificity [62]. Given our finding that certain sub-groups reported diametrically opposite changes in technology use when psychotic symptoms were high (a third increased use; a third decreased), analyses of such passively collected data must take into account variation between individuals.

Only a minority of participants owned a fitness tracker or smartwatch. Unlike smartphone-based digital health tools, it seems unlikely that passive sensing using wearables would be widely adopted, at present, without a mechanism to provide users with such devices. Nevertheless, it is likely that wearables will become cheaper and more widely owned in future; hence, research testing the potential value of passive sensing using wearables is warranted. As well as large-scale cohort studies examining clinical correlates of specific types of passively gathered data [62], studies gathering detailed views from stakeholders will be important to accelerate future implementation [55]. It will be important to weigh up potentially competing considerations such as the quality of data provided by specific wearables (research-grade devices provide the highest data quality [63]) versus acceptability of the device to people with psychosis (consumer grade devices may be most acceptable [64]). Finally, we recommend that research studies or clinics testing passive sensing using wearable devices should ensure they have a dedicated budget to provide wearable devices to participants to allow more representative samples to be included.

### Limitations

There were some limitations. First, some participants were recruited in the context of taking part in a digital health trial, and others took part in response to online advertisements; this self-selected sample may have been more familiar, interested and comfortable with using digital tools than is typical; additionally, those who self-select to take part in research are often more engaged with services. Nevertheless, the Actissist2 participants were recruited from across Early Intervention Services, inclusion criteria for taking part were intentionally wide, and participants were lent a phone to use in the study if they did not have one themselves. The demographic makeup of the Actissist2 sample was characteristic of a psychosis sample recruited from the North West of England. Compared to the online sample, the Actissist2 sample was more male, more ethnically diverse, and more likely to be single than the online sample. Second, participants from the online sample self-reported their diagnosis. Although commonly used as a recruitment strategy, this may mean that some participants who took part did not meet full diagnostic criteria for a psychosis-related disorder. Third, the survey closed in September 2020. As availability of digital devices becomes more widespread, and in light of the Covid-19 pandemic which accelerated digital tool use, data presented here might not fully reflect how individuals with psychosis currently access and use digital tools. Finally, the current survey did not gather detailed information on the acceptability of newer developments such as passive sensing using smartphones/wearables. Our research group is currently gathering up-to-date mixed methods data addressing this question.

## Conclusions

We found high mobile phone ownership rates and high levels of interest in using a mental health app. Participants thought using an app could increase their understanding of psychosis generally, and of their own symptoms. They valued the flexibility of digital tools in enabling access to mental health support anytime and anywhere. Nevertheless, some barriers to phone ownership (e.g. cost, paranoia) and mental health app use (e.g. forgetting, lack of motivation, security concerns) were reported.

### Supplementary Information

**Additional file 1.** Survey.

## Data Availability

The datasets used and/or analysed during the current study available from the corresponding author on reasonable request.

## Abbreviations

SMI: Severe mental health problems
US: United States
UK: United Kingdom
CI: Confidence interval

## References

[1] Zhang X, Lewis S, Firth J, Chen X, Bucci S (2021). Digital mental health in China: a systematic review. Psychol Med.

[2] Chivilgina O, Wangmo T, Elger BS, Heinrich T, Jotterand F (2020). mHealth for schizophrenia spectrum disorders management: a systematic review. Int J Soc Psychiatry.

[3] Carter H, Araya R, Anjur K, Deng D, Naslund JA (2021). The emergence of digital mental health in low-income and middle-income countries: A review of recent advances and implications for the treatment and prevention of mental disorders. J Psychiatr Res.

[4] Spanhel K, Balci S, Feldhahn F, Bengel J, Baumeister H, Sander LB (2021). Cultural adaptation of internet- and mobile-based interventions for mental disorders: a systematic review. npj Digit Med.

[5] Jameel L, Valmaggia L, Barnes G, Cella M (2022). mHealth technology to assess, monitor and treat daily functioning difficulties in people with severe mental illness: a systematic review. J Psychiatr Res.

[6] Eisner E, Bucci S, Berry N, Emsley R, Barrowclough C, Drake RJ (2019). Feasibility of using a smartphone app to assess early signs, basic symptoms and psychotic symptoms over six months: a preliminary report. Schizophr Res.

[7] Eisner E, Drake RJ, Berry N, Barrowclough C, Emsley R, Machin M (2019). Development and long-term acceptability of ExPRESS, a mobile phone app to monitor basic symptoms and early signs of psychosis relapse. JMIR Mhealth Uhealth.

[8] Gumley AI, Bradstreet S, Ainsworth J, Allan S, Alvarez-Jimenez M, Birchwood M (2022). Digital smartphone intervention to recognise and manage early warning signs in schizophrenia to prevent relapse: the EMPOWER feasibility cluster RCT. Health Technol Assess.

[9] Gumley AI, Bradstreet S, Ainsworth J, Allan S, Alvarez-Jimenez M, Aucott L (2022). The EMPOWER blended digital intervention for relapse prevention in schizophrenia: a feasibility cluster randomised controlled trial in Scotland and Australia. Lancet Psychiatry.

[10] Garety P, Ward T, Kuipers E, Hardy A, Emsley R, James K (2021). Effects of slowMo, a blended digital therapy targeting reasoning, on paranoia among people with psychosis: a randomized clinical trial. JAMA Psychiat.

[11] Clarke S, Hanna D, Mulholland C, Shannon C, Urquhart C (2019). A systematic review and meta-analysis of digital health technologies effects on psychotic symptoms in adults with psychosis. Psychosis.

[12] Spanakis P, Peckham E, Gilbody S, Mathers A, Shiers D (2021). The digital divide: amplifying health inequalities for people with severe mental illness in the time of COVID-19. Br J Psychiatry.

[13] Borghouts J, Eikey E, Mark G, De Leon C, Schueller SM, Schneider M (2021). Barriers to and facilitators of user engagement with digital mental health interventions: systematic review. J Med Internet Res.

[14] Firth J, Cotter J, Torous J, Bucci S, Firth JA, Yung AR (2016). Mobile phone ownership and endorsement of “mhealth” among people with psychosis: a meta-analysis of cross-sectional studies. Schizophr Bull.

[15] Gay K, Torous J, Joseph A, Pandya A, Duckworth K (2016). Digital technology use among individuals with schizophrenia: results of an online survey. JMIR Mental Health.

[16] Robotham D, Satkunanathan S, Doughty L, Wykes T (2016). Do we still have a digital divide in mental health? A five-year survey follow-up. J Med Internet Res.

[17] Bonet L, Llacer B, Hernandez-Viadel M, Arce D, Blanquer I, Canete C (2018). Differences in the use and opinions about new ehealth technologies among patients with psychosis: structured questionnaire. JMIR Ment Health.

[18] Simões de Almeida R, Sousa T, Marques A, Queirós C (2018). Patients’ perspectives about the design of a mobile application for psychotic disorders. Psychol Comm Health.

[19] Torous J, Wisniewski H, Liu G, Keshavan M (2018). Mental health mobile phone app usage, concerns, and benefits among psychiatric outpatients: comparative survey study. JMIR Mental Health.

[20] Wong KTG, Liu D, Balzan R, King D, Galletly C (2020). Smartphone and internet access and utilization by people with Schizophrenia in South Australia: quantitative survey study. JMIR Mental Health.

[21] Watson A, Mellotte H, Hardy A, Peters E, Keen N, Kane F (2021). The digital divide: factors impacting on uptake of remote therapy in a South London psychological therapy service for people with psychosis. J Ment Health.

[22] Luther L, Buck BE, Fischer MA, Johnson-Kwochka AV, Coffin G, Salyers MP (2022). Examining potential barriers to mhealth implementation and engagement in schizophrenia: phone ownership and symptom severity. J Technol Behav Sci.

[23] Lal S, Abdel-Baki A, Sujanani S, Bourbeau F, Sahed I, Whitehead J (2020). Perspectives of young adults on receiving telepsychiatry services in an urban early intervention program for first-episode psychosis: a cross-sectional. Descript Survey Stud Front Psychiatry.

[24] Naslund JA, Aschbrenner KA (2021). Technology use and interest in digital apps for mental health promotion and lifestyle intervention among young adults with serious mental illness. J Affect Disord Rep.

[25] Harris PA, Taylor R, Thielke R, Payne J, Gonzalez N, Conde JG (2009). Research electronic data capture (REDCap) - A metadata-driven methodology and workflow process for providing translational research informatics support. J Biomed Inform.

[26] Harris PA, Taylor R, Minor BL, Elliott V, Fernandez M, O’Neal L (2019). The REDCap consortium: building an international community of software platform partners. J Biomed Inform.

[27] Ben-Zeev D, Davis KE, Kaiser S, Krzsos I, Drake RE (2013). Mobile technologies among people with serious mental illness: opportunities for future services. Adm Policy Ment Health.

[28] Naslund JA, Aschbrenner KA, Bartels SJ (2016). How people with serious mental illness use smartphones, mobile apps, and social media. Psychiatr Rehabil J.

[29] Torous J, Chan SR, Yee-Marie Tan S, Behrens J, Mathew I, Conrad EJ (2014). Patient smartphone ownership and interest in mobile apps to monitor symptoms of mental health conditions: a survey in four geographically distinct psychiatric clinics. JMIR Mental Health.

[30] Gipson S, Torous J, Boland R, Conrad E (2017). Mobile phone use in psychiatry residents in the United States: multisite cross-sectional survey study. JMIR Mhealth Uhealth.

[31] Young KS (1998). Internet addiction: the emergence of a new clinical disorder. Cyberpsychol Behav.

[32] Matthews M, Murnane E, Snyder J, Guha S, Chang P, Doherty G (2017). The double-edged sword: a mixed methods study of the interplay between bipolar disorder and technology use. Comput Hum Behav.

[33] Berry N, Lobban F, Bucci S (2019). A qualitative exploration of service user views about using digital health interventions for self-management in severe mental health problems. BMC Psychiatry.

[34] Berry N, Bucci S, Lobban F (2017). Use of the internet and mobile phones for self-management of severe mental health problems: qualitative study of staff views. JMIR Mental Health.

[35] Bucci S, Berry N, Morris R, Berry K, Haddock G, Lewis S (2019). “They are not hard-to-reach clients. We have just got hard-to-reach services.” Staff views of digital health tools in specialist mental health services. Front Psychiatry.

[36] Bucci S, Morris R, Berry K, Berry N, Haddock G, Barrowclough C (2018). Early psychosis service user views on digital technology: qualitative analysis. JMIR Ment Health.

[37] Field AP (2009). Discovering statistics using SPSS: and sex, drugs and rock “n” roll.

[38] StataCorp (2015). Stata statistical software.

[39] Asuzu K, Rosenthal MZ (2021). Mobile device use among inpatients on a psychiatric unit: a preliminary study. Psychiatry Res.

[40] Deb KS, Sood M, Chadda R, Verma R, Kumar S, Ganesh R (2018). Is India ready for mental health apps (MHApps)? A quantitative-qualitative exploration of caregivers’ perspective on smartphone-based solutions for managing severe mental illnesses in low resource settings. PLoS ONE.

[41] Fortuna KL, Aschbrenner KA, Brooks J, Lohman MC, Salzer M, Walker R (2018). Smartphone ownership, use, and willingness to use smartphones to provide peer-delivered services: results from a national online survey. Psychiatr Q.

[42] Iliescu R, Kumaravel A, Smurawska L, Torous J, Keshavan M (2021). Smartphone ownership and use of mental health applications by psychiatric inpatients. Psychiatry Res.

[43] Klee A, Stacy M, Rosenheck R, Harkness L, Tsai J (2016). Interest in technology-based therapies hampered by access: a survey of veterans with serious mental illnesses. Psychiatr Rehabil J.

[44] Noel VA, Acquilano SC, Carpenter-Song E, Drake RE (2019). Use of mobile and computer devices to support recovery in people with serious mental illness: survey study. JMIR Ment Health.

[45] Abu Rahal Z, Vadas L, Manor I, Bloch B, Avital A (2018). Use of information and communication technologies among individuals with and without serious mental illness. Psychiatry Res.

[46] Tobitt S, Percival R (2019). Switched on or switched off? A survey of mobile, computer and Internet use in a community mental health rehabilitation sample. J Ment Health.

[47] Bell IH, Thompson A, Valentine L, Adams S, Alvarez-Jimenez M, Nicholas J (2022). Ownership, use of, and interest in digital mental health technologies among clinicians and young people across a spectrum of clinical care needs: cross-sectional survey. JMIR Ment Health.

[48] Pew Research Centre. Demographics of mobile device ownership and adoption in the United States. 2021. https://www.pewresearch.org/internet/fact-sheet/mobile/. Accessed 6 Oct 2023.

[49] Deloitte. Mobile consumer survey 2019 – theme 2. Wearables are on the rise. 2019. https://www2.deloitte.com/be/en/pages/technology-media-and-telecommunications/topics/mobile-consumer-survey-2019/wearables.html. Accessed 6 Oct 2023.

[50] Shpigelman CN, Tal A, Zisman-Ilani Y (2021). Digital community inclusion of individuals with serious mental illness: a national survey to map digital technology use and community participation patterns in the digital era. JMIR Ment Health.

[51] Marwaha S, Johnson S (2004). Schizophrenia and employment. Soc Psychiatry Psychiatr Epidemiol.

[52] Świtaj P, Anczewska M, Chrostek A, Sabariego C, Cieza A, Bickenbach J (2012). Disability and schizophrenia: a systematic review of experienced psychosocial difficulties. BMC Psychiatry.

[53] Kennedy JL, Altar CA, Taylor DL, Degtiar I, Hornberger JC (2014). The social and economic burden of treatment-resistant schizophrenia: a systematic literature review. Int Clin Psychopharmacol.

[54] Villagonzalo K-A, Arnold C, Foley F, Farhall J, Rossell SL, Thomas N (2019). Predictors of overall and mental health-related internet use in adults with psychosis. Psychiatry Res.

[55] Aref-Adib G, McCloud T, Ross J, O’Hanlon P, Appleton V, Rowe S (2019). Factors affecting implementation of digital health interventions for people with psychosis or bipolar disorder, and their family and friends: a systematic review. Lancet Psychiatry.

[56] Berry N, Emsley R, Lobban F, Bucci S (2018). Social media and its relationship with mood, self-esteem and paranoia in psychosis. Acta Psychiatr Scand.

[57] Huo J, Desai R, Hong Y-R, Turner K, Mainous AG, Bian J (2019). Use of social media in health communication: findings from the health information national trends survey 2013, 2014, and 2017. Cancer Contr.

[58] Chakravorty T (2022). The impact of financial crises on mental health: review. Phy.

[59] Deloitte. Digital consumer trends 2022 devices: growth on pause, for now. 2022. https://www2.deloitte.com/content/dam/Deloitte/uk/Documents/technology-media-telecommunications/deloitte-uk-digital-consumer-trends-2022-device-usage.pdf. Accessed 6 Oct 2023.

[60] Sheikh M, Qassem M, Kyriacou PA (2021). Wearable, environmental, and smartphone-based passive sensing for mental health monitoring. Front Digit Health.

[61] Torous J, Wisniewski H, Bird B, Carpenter E, David G, Elejalde E (2019). Creating a digital health smartphone app and digital phenotyping platform for mental health and diverse healthcare needs: an interdisciplinary and collaborative approach. J Technol Behav Sci.

[62] Benoit J, Onyeaka H, Keshavan M, Torous J (2020). Systematic review of digital phenotyping and machine learning in psychosis spectrum illnesses. Harv Rev Psychiatry.

[63] Cho S, Ensari I, Weng C, Kahn MG, Natarajan K (2021). Factors affecting the quality of person-generated wearable device data and associated challenges: rapid systematic review. JMIR Mhealth Uhealth.

[64] Matcham F, di San Pietro CB, Bulgari V, De Girolamo G, Dobson R, Eriksson H (2019). Remote assessment of disease and relapse in major depressive disorder (RADAR-MDD): a multi-centre prospective cohort study protocol. BMC Psychiatry.

