# Supplementary material for: Digital tools to support mental health: a survey study in psychosis
Source: BMC Psychiatry. 2023 Oct 7;23:726. doi: 10.1186/s12888-023-05114-y (PMC10559432; doi:10.1186/s12888-023-05114-y)
Supplement: Supplementary file 1 — Additional file 1. Survey. [file 12888_2023_5114_MOESM1_ESM.docx]

**EMIS (eHealth and mHealth Interest Survey) – Version 4, 04.06.18**

Participant ID: ___________ Date of completion: ______________

**Technology ownership/use questions**

1. Which of the following do you own or have access to? (please circle to indicate):

| Mobile phone | Yes (I own one) | Yes (but it belongs to someone else) | No |
| --- | --- | --- | --- |
| Smartphone | Yes (I own one) | Yes (but it belongs to someone else) | No |
| Internet | Yes (I pay) | Yes (but someone else pays) | No |
| Laptop or desktop computer | Yes (I own one) | Yes (but it belongs to someone else) | No |
| Wearable fitness tracker (e.g. Fitbit) | Yes (I own one) | Yes (but it belongs to someone else) | No |
| Smartwatch (e.g. Apple watch) | Yes (I own one) | Yes (but it belongs to someone else) | No |

2. What operating system does your smartphone use?

iOS (Apple Iphone) Android Don’t know

NA (I do not use a smartphone)

3. How do you pay for your phone use?

Monthly contract (phone plus sim) Pay as you go

Monthly contract (sim only) Someone else pays

4. What features do you use on your phone (please tick all that apply):

Phone calls Alarm

Text messages Calendar

Email Radio

Internet browser Music

Smartphone apps Camera

5. How often do you use the following? (please circle to indicate)

| Mobile phone | Multiple times a day | Once a day | A few times a week | Once a week | A few times a month | Once a month | Less often | N/A I do not use this |
| --- | --- | --- | --- | --- | --- | --- | --- | --- |
| Smartphone | Multiple times a day | Once a day | A few times a week | Once a week | A few times a month | Once a month | Less often | N/A I do not use this |
| Smartphone apps | Multiple times a day | Once a day | A few times a week | Once a week | A few times a month | Once a month | Less often | N/A I do not use this |
| Internet via mobile phone/tablet | Multiple times a day | Once a day | A few times a week | Once a week | A few times a month | Once a month | Less often | N/A I do not use this |
| Internet via computer/laptop | Multiple times a day | Once a day | A few times a week | Once a week | A few times a month | Once a month | Less often | N/A I do not use this |
| Social media (e.g. Facebook, Twitter) | Multiple times a day | Once a day | A few times a week | Once a week | A few times a month | Once a month | Less often | N/A I do not use this |
| Smartwatch (e.g. Apple watch) | Multiple times a day | Once a day | A few times a week | Once a week | A few times a month | Once a month | Less often | N/A I do not use this |
| Fitness tracker (e.g. FitBit) | Multiple times a day | Once a day | A few times a week | Once a week | A few times a month | Once a month | Less often | N/A I do not use this |
| Laptop computer | Multiple times a day | Once a day | A few times a week | Once a week | A few times a month | Once a month | Less often | N/A I do not use this |
| Desktop computer | Multiple times a day | Once a day | A few times a week | Once a week | A few times a month | Once a month | Less often | N/A I do not use this |
| Tablet computer | Multiple times a day | Once a day | A few times a week | Once a week | A few times a month | Once a month | Less often | N/A I do not use this |

**Mobile phone apps**

6. How often do you use the following mobile phone apps? (please circle to indiciate)

| Instant messaging apps (e.g. WhatsApp, Facebook Messenger) | Multiple times a day | Once a day | A few times a week | Once a week | A few times a month | Once a month | Less often | I have downloaded this type of app, but never used it | I have never downloaded this type of app |
| --- | --- | --- | --- | --- | --- | --- | --- | --- | --- |
| Social media apps (e.g. Facebook, Twitter) | Multiple times a day | Once a day | A few times a week | Once a week | A few times a month | Once a month | Less often | I have downloaded this type of app, but never used it | I have never downloaded this type of app |
| Entertainment apps (e.g. BBC IPlayer, Capital FM, All 4) | Multiple times a day | Once a day | A few times a week | Once a week | A few times a month | Once a month | Less often | I have downloaded this type of app, but never used it | I have never downloaded this type of app |
| Video calling apps (e.g. FaceTime, Skype) | Multiple times a day | Once a day | A few times a week | Once a week | A few times a month | Once a month | Less often | I have downloaded this type of app, but never used it | I have never downloaded this type of app |
| Gaming apps (e.g. Brain Training, Candy Crush) | Multiple times a day | Once a day | A few times a week | Once a week | A few times a month | Once a month | Less often | I have downloaded this type of app, but never used it | I have never downloaded this type of app |
| Exercise apps (e.g. Couch to 5k) | Multiple times a day | Once a day | A few times a week | Once a week | A few times a month | Once a month | Less often | I have downloaded this type of app, but never used it | I have never downloaded this type of app |
| Diet/healthy eating apps (e.g. Change4Life, MyFitnessPal) | Multiple times a day | Once a day | A few times a week | Once a week | A few times a month | Once a month | Less often | I have downloaded this type of app, but never used it | I have never downloaded this type of app |
| Quit smoking apps (e.g. NHS Smoke free) | Multiple times a day | Once a day | A few times a week | Once a week | A few times a month | Once a month | Less often | I have downloaded this type of app, but never used it | I have never downloaded this type of app |
| Mindfulness/ meditation apps (e.g. Headspace) | Multiple times a day | Once a day | A few times a week | Once a week | A few times a month | Once a month | Less often | I have downloaded this type of app, but never used it | I have never downloaded this type of app |
| Mental health apps (e.g. Silvercloud, Catch It) | Multiple times a day | Once a day | A few times a week | Once a week | A few times a month | Once a month | Less often | I have downloaded this type of app, but never used it | I have never downloaded this type of app |

7. If you have ever used mental health/wellbeing/mindfulness app(s), please can you indicate below which apps these were, whether you currently use them, how often you use them and how helpful you found them (1 = unhelpful; 4 = very helpful)? If you cannot remember the name of the app, perhaps check your phone or alternatively write a description of what the app does.

| App name | Current or past use *(circle to indicate)* | | Frequency of use (1=multiple times a day; 2=once a day; 3=a few times a week; 4=once a week; 5=a few times a month; 6=less often) | | | | | | Perceived helpfulness (1= unhelpful - it made me feel worse; 2 = neutral - it didn’t help but it didn’t make me feel worse; 3 = helpful; 4 = very helpful) | | | |
| --- | --- | --- | --- | --- | --- | --- | --- | --- | --- | --- | --- | --- |
| App 1 (please specify)  …………………………  ………………………… | Current | Past | 1 | 2 | 3 | 4 | 5 | 6 | 1 | 2 | 3 | 4 |
| App 2 (please specify)  …………………………  ………………………… | Current | Past | 1 | 2 | 3 | 4 | 5 | 6 | 1 | 2 | 3 | 4 |
| App 3 (please specify)  …………………………  ………………………… | Current | Past | 1 | 2 | 3 | 4 | 5 | 6 | 1 | 2 | 3 | 4 |
| App 4 (please specify)  …………………………  ………………………… | Current | Past | 1 | 2 | 3 | 4 | 5 | 6 | 1 | 2 | 3 | 4 |
| App 5 (please specify)  ………………………… | Current | Past | 1 | 2 | 3 | 4 | 5 | 6 | 1 | 2 | 3 | 4 |

N/A (I have never used a mental health app)

8. What are the barriers you have faced, if any, to being able to own or use a mobile phone? (please tick all that apply):

I struggle to afford to own and/or use a mobile phone

I’m not interested in mobile phones

I don’t need to use a mobile phone

I keep losing or damaging mobile phones

I don’t know how to use a mobile phone

I don’t know how to use certain mobile phone features (e.g. smartphone apps)

I feel paranoid or suspicious about mobile phones

Not applicable (there are no barriers for me)

Other (please state) ………………………

9. To what extent do you agree or disagree with the following statements about mobile phones (1 = strongly disagree; 5 = strongly agree)? (please circle to indicate)

| Question | Strongly disagree | Disagree | Neutral | Agree | Strongly agree |
| --- | --- | --- | --- | --- | --- |
| Using a mobile phone makes me depressed or anxious | 1 | 2 | 3 | 4 | 5 |
| Using a mobile phone makes me happy | 1 | 2 | 3 | 4 | 5 |
| Using a mobile phone makes me feel paranoid or suspicious | 1 | 2 | 3 | 4 | 5 |
| Using a mobile phone makes voices worse | 1 | 2 | 3 | 4 | 5 |
| Using a mobile phone makes voices better | 1 | 2 | 3 | 4 | 5 |
| I worry people with find out about my mental health if I use a mobile phone | 1 | 2 | 3 | 4 | 5 |
| Using a mobile phone helps me socialise with people outside my home | 1 | 2 | 3 | 4 | 5 |
| Using a mobile phone helps me to feel connected | 1 | 2 | 3 | 4 | 5 |
| Using a mobile phone makes me compare myself with others | 1 | 2 | 3 | 4 | 5 |
| Using a mobile phone helps my overall mental health and wellbeing | 1 | 2 | 3 | 4 | 5 |
| Using a mobile phone is unhelpful for my overall mental health and wellbeing | 1 | 2 | 3 | 4 | 5 |

10. Have you ever shared any of the following information with a member of your care team?

a. Information you have found online about psychiatric medications

Yes No

b. Information you have found online about psychological therapy

Yes No

**Social media**

11. Which social media website(s)/apps(s) do you access? (please circle to indicate)

| Facebook | Multiple times a day | Daily | A few times a week | Once a week | A few times a month | Once a month | Less often | Never |
| --- | --- | --- | --- | --- | --- | --- | --- | --- |
| Twitter | Multiple times a day | Daily | A few times a week | Once a week | A few times a month | Once a month | Less often | Never |
| Instagram | Multiple times a day | Daily | A few times a week | Once a week | A few times a month | Once a month | Less often | Never |
| Snapchat | Multiple times a day | Daily | A few times a week | Once a week | A few times a month | Once a month | Less often | Never |
| LinkedIn | Multiple times a day | Daily | A few times a week | Once a week | A few times a month | Once a month | Less often | Never |
| MySpace | Multiple times a day | Daily | A few times a week | Once a week | A few times a month | Once a month | Less often | Never |
| Google Plus | Multiple times a day | Daily | A few times a week | Once a week | A few times a month | Once a month | Less often | Never |

12. Have you ever posted information about your physical health on a social media website/app? (please tick to indicate)

Yes No N/A (I have never used social media)

13. Have you ever posted information about your mental health on a social media website app? (please tick to indicate)

Yes No N/A (I have never used social media)

14. To what extent do you agree or disagree with the following statements? (please circle to indicate)

| Question | Strongly disagree | Disagree | Neutral | Agree | Strongly agree |
| --- | --- | --- | --- | --- | --- |
| Using social media makes me feel depressed or anxious | 1 | 2 | 3 | 4 | 5 |
| Using social media makes me happy | 1 | 2 | 3 | 4 | 5 |
| Using social media makes me feel paranoid or suspicious | 1 | 2 | 3 | 4 | 5 |
| Using social media makes voices worse | 1 | 2 | 3 | 4 | 5 |
| Using social media makes voices better | 1 | 2 | 3 | 4 | 5 |
| I worry that people will find out about my mental health if I use social media | 1 | 2 | 3 | 4 | 5 |
| Using social media helps me interact with friends and/or family | 1 | 2 | 3 | 4 | 5 |
| Using social media helps me feel connected | 1 | 2 | 3 | 4 | 5 |
| Using social media helps me socialise more with people outside my home | 1 | 2 | 3 | 4 | 5 |
| Using social media makes me compare myself with others | 1 | 2 | 3 | 4 | 5 |
| I would participate in a social media group of others with psychosis | 1 | 2 | 3 | 4 | 5 |
| Using a social media helps my overall mental health and wellbeing | 1 | 2 | 3 | 4 | 5 |
| Using social media is unhelpful for my overall mental health and wellbeing | 1 | 2 | 3 | 4 | 5 |

**General technology use**

15. Aside from telephone calls, how frequently do you use a computer, mobile phone, smartwatch or tablet computer to do the following? (please circle to indicate):

| Identify coping strategies | Very often | Often | Sometimes | Rarely | Never |
| --- | --- | --- | --- | --- | --- |
| Monitor symptoms | Very often | Often | Sometimes | Rarely | Never |
| Develop relationships with other individuals who have a lived experience related to mental health problems | Very often | Often | Sometimes | Rarely | Never |
| Provide support to others | Very often | Often | Sometimes | Rarely | Never |
| Set alarms/reminders to help with medication management | Very often | Often | Sometimes | Rarely | Never |
| Use calendar or set alerts/reminders for appointments | Very often | Often | Sometimes | Rarely | Never |
| Find information about mental health problems | Very often | Often | Sometimes | Rarely | Never |
| Find information about physical health problems | Very often | Often | Sometimes | Rarely | Never |
| Listen to music or audio files to help block or manage voices | Very often | Often | Sometimes | Rarely | Never |
| Record voices or sounds that I hear that others do not | Very often | Often | Sometimes | Rarely | Never |
| Take photos of objects or people that I see that others do not | Very often | Often | Sometimes | Rarely | Never |

16. Please answer the following questions related to how you use technology (please circle to indicate)

| How often do you find yourself saying “just a few more minutes” when online or using your mobile phone? | Always | Often | Frequently | Occasionally | Rarely | Does not apply |
| --- | --- | --- | --- | --- | --- | --- |
| How often do you try to cut down the amount of time you spend online or using your mobile phone and fail? | Always | Often | Frequently | Occasionally | Rarely | Does not apply |
| How often do you lose sleep due to late-night technology use? | Always | Often | Frequently | Occasionally | Rarely | Does not apply |
| How often do you find that you stay online or on your mobile phone longer than you intended? | Always | Often | Frequently | Occasionally | Rarely | Does not apply |

17. To what extent do you agree the following statements regarding your technology use (1 = strongly disagree; 5 strongly agree)

| Question | Strongly disagree | Disagree | Neither agree nor disagree | Agree | Strongly agree |
| --- | --- | --- | --- | --- | --- |
| My technology use generally remains constant | 1 | 2 | 3 | 4 | 5 |
| I tend to use technology more when I am experiencing symptoms of psychosis | 1 | 2 | 3 | 4 | 5 |
| I tend to use technology less when I am experiencing symptoms of psychosis | 1 | 2 | 3 | 4 | 5 |
| I tend to use technology more when my mood is low | 1 | 2 | 3 | 4 | 5 |
| I tend to use technology less when my mood is low | 1 | 2 | 3 | 4 | 5 |

18. To what extent do you agree or disagree with the following statements? (please circle to indicate)

| Question | Strongly disagree | Disagree | Neutral | Agree | Strongly agree |
| --- | --- | --- | --- | --- | --- |
| I would like to receive text messages from my care team to remind me about appointments | 1 | 2 | 3 | 4 | 5 |
| I would like a smartphone app to alert me with appointments | 1 | 2 | 3 | 4 | 5 |
| I would like to receive text messages from my care team to remind me about taking my medication | 1 | 2 | 3 | 4 | 5 |
| I would like a smartphone app to alert me with medication reminders | 1 | 2 | 3 | 4 | 5 |
| I would like to receive text messages from my care team to ask me about symptoms, medication side effects or other problems | 1 | 2 | 3 | 4 | 5 |

**Mobile phone apps**

19. Would you be willing to use a mobile phone app to keep a record of your symptoms over time? (please tick to indicate)

Yes No *(if no skip to question 22)*

20. Would you be willing for members of your care team (e.g. care coordinator, psychiatrist) to receive the symptom information you enter into a mobile phone app? (please tick to indicate)

Yes (I would be happy for the information to be automatically transferred to my care team)

Yes (I would be happy for my care team to receive the information, but I would want to choose what information is transferred)

Yes (I would be happy for my care team to receive the information, but I would want to take it to appointments myself to show them)

No (I would not want my care team to see the symptom information I enter)

21. Would you prefer to receive reminders to answer questions on an app about your mental health or complete questions when you feel like it?

I would like to receive reminders

I would like to complete assessments when I choose

I would like a combination

22. How much would you be willing to pay for a mental health app?

Not willing to pay for a mental health app

Willing to pay up to 99 pence for a mental health app

Willing to pay more than 99 pence for a mental health app

23. If you were offered a mobile phone app for your mental health, do you think you would try it? (please tick to indicate)

Yes No Unsure

24. If you were offered a mobile phone app, what other support do you think you would like to receive? (please tick to indicate)

I would be happy to use the app on its own with no other mental health support

I would want to use the app in conjunction remote mental health support (e.g. telephone, video-calling)

I would want to use the app in conjunction with face-to-face support

I would not want to use a mental health app

25. To what extent do you agree or disagree (1 = strongly disagree; 5 = strongly agree) that the following barriers would affect the likelihood of you using a mental health app? (please tick all that apply)

| Barrier | Strongly disagree | Disagree | Neutral | Agree | Strongly agree |
| --- | --- | --- | --- | --- | --- |
| Smartphone handset costs | 1 | 2 | 3 | 4 | 5 |
| Smartphone data costs | 1 | 2 | 3 | 4 | 5 |
| Poor storage for apps on smartphone | 1 | 2 | 3 | 4 | 5 |
| Smartphone technology skills | 1 | 2 | 3 | 4 | 5 |
| Reading difficulties | 1 | 2 | 3 | 4 | 5 |
| Physical problems (e.g. poor eyesight hand tremors) | 1 | 2 | 3 | 4 | 5 |
| Lack of motivation | 1 | 2 | 3 | 4 | 5 |
| Forgetting to use the app | 1 | 2 | 3 | 4 | 5 |
| Concerns about how helpful a mental health app would be | 1 | 2 | 3 | 4 | 5 |
| Concerns that a mental health app would be used as an excuse to replace face-to-face support | 1 | 2 | 3 | 4 | 5 |
| Concerns about the where the information I put in the app would go/who would get access | 1 | 2 | 3 | 4 | 5 |
| Concerns that the app could be hacked | 1 | 2 | 3 | 4 | 5 |
| Feeling suspicious or paranoid about mobile phones in general | 1 | 2 | 3 | 4 | 5 |
| Feeling suspicious or paranoid about the app specifically | 1 | 2 | 3 | 4 | 5 |
| Already able to manage mental health - do not need a mental health app | 1 | 2 | 3 | 4 | 5 |
| Concerns about focussing too much on symptoms | 1 | 2 | 3 | 4 | 5 |
| Concerns about being unable to personalise/tailor mental health apps | 1 | 2 | 3 | 4 | 5 |

26. Please list any other barriers that you can think of that would prevent you from using a mental health app.

………………………………………………………………………………………………………………………………………………………………………………………………………………………………………………………………………………………………………………………………………………………………………

27. To what extent do you agree or disagree (1 = strongly disagree; 5 = strongly agree) that the following are potential advantages of mental health apps? (please tick all that apply)

|  | Strongly disagree | Disagree | Neutral | Agree | Strongly agree |
| --- | --- | --- | --- | --- | --- |
| Ability to access an app at any time | 1 | 2 | 3 | 4 | 5 |
| Ability to access an app in any location | 1 | 2 | 3 | 4 | 5 |
| Opportunity to take control over mental health | 1 | 2 | 3 | 4 | 5 |
| Opportunity to learn more about psychosis | 1 | 2 | 3 | 4 | 5 |
| Opportunity to increase understanding about own symptoms and experiences | 1 | 2 | 3 | 4 | 5 |
| More comfortable providing honest responses to an anonymous/faceless device | 1 | 2 | 3 | 4 | 5 |
| Ability to start and stop “sessions” whenever I wish | 1 | 2 | 3 | 4 | 5 |
| Opportunity to record, and reflect back on, symptoms and experiences over time | 1 | 2 | 3 | 4 | 5 |
| Opportunity to take up-to-date records of symptoms and experiences to clinicians | 1 | 2 | 3 | 4 | 5 |
| Opportunity to identify triggers and patterns | 1 | 2 | 3 | 4 | 5 |
| Cost-effective alternative to face-to-face support | 1 | 2 | 3 | 4 | 5 |
| Opportunity to connect with others using the app | 1 | 2 | 3 | 4 | 5 |
| Potential for staff to intervene early if increases in symptoms are identified | 1 | 2 | 3 | 4 | 5 |
| Increased privacy in comparison to paper-based symptom monitoring or therapy materials | 1 | 2 | 3 | 4 | 5 |
| Less stigmatising than attending therapy | 1 | 2 | 3 | 4 | 5 |
| Having a mental health app available is normalising and de-shaming | 1 | 2 | 3 | 4 | 5 |

28. Please list any other reasons why you might want to use a mental health app?

………………………………………………………………………………………………………………………………………………………………………………………………………………………………………………………………………………………………………………………………………………………………………

29. Do you have any ideas for any other content you would like to see in a mental health app?

………………………………………………………………………………………………………………………………………………………………………………………………………………………………………………………………………………………………………………………………………………………………………

30. Please indicate how much you agree or disagree with the following statements (please circle)

| Question | Strongly disagree | Disagree | Neither agree nor disagree | Agree | Strongly agree |
| --- | --- | --- | --- | --- | --- |
| I am enthusiastic about electronics and digital devices | 1 | 2 | 3 | 4 | 5 |
| I frequently look for new software or apps | 1 | 2 | 3 | 4 | 5 |
| My friends would describe me as “into” the latest technology | 1 | 2 | 3 | 4 | 5 |
| For me, technology is frustrating | 1 | 2 | 3 | 4 | 5 |

31. Some researchers are now trialling “digital pills” for psychosis. These pills transmit a signal to a wearable patch, which then records whether someone has consumed medication using a smartphone app. This allows service users to track whether they have taken their medication and receive reminders if they have forgotten. Service users would also have the option to allow their care team or support network (e.g. a carer, family member or close friend) access to the smartphone app to track medication use.

a. How acceptable do you think “digital pills” are for psychosis? (please tick to indicate):

Acceptable Not acceptable Unsure

b. How helpful do you think “digital pills” could be for people with psychosis? (please tick to indicate):

Helpful Unhelpful Unsure

c. How likely do you think it would be that you would use “digital pills” if you were offered them? (please tick to indicate):

Not at all likely Unlikely Likely Very likely Unsure

N/A (I do not take medication)

d. Who would you authorise to track your “digital pill” consumption? (please select all that apply):

Only me (I would not want any other person to track my “digital pill” consumption)

My care coordinator/CPN

My psychiatrist

My psychologist

My partner

Selected family member(s)

Selected friend(s)

N/A (I would not want to use “digital pills”/I do not take medication)

32. Have you ever communicated with an artificial agent (e.g. IPhone Siri, Microsoft Cortana, Amazon Alexa) about your mental health? (please tick all that apply)

No

Yes - to find out information about local services

Yes - to feel like I’ve spoken to someone about how I am feeling

Yes - to find mental health resources

Yes - other (please state) ……………………….

33. Thinking about the last time you communicated with the artificial agent, how helpful did you find the response?

Unhelpful - the response was upsetting

Unhelpful - the response was not relevant

Helpful

N/A (I have never communicated with an artificial agent)
